# Yeast extracts from different manufacturers and supplementation of amino acids and micro elements reveal a remarkable impact on alginate production by *A. vinelandii* ATCC9046

**DOI:** 10.1186/s12934-023-02112-3

**Published:** 2023-05-11

**Authors:** Sarah Sparviero, Max Daniel Dicke, Tobias M. Rosch, Tania Castillo, Holjes Salgado-Lugo, Enrique Galindo, Carlos Peña, Jochen Büchs

**Affiliations:** 1 Aachener Verfahrenstechnik – Chair of Biochemical Engineering, RWTH Aachen University, Bldg. NGP², Forckenbeckstr. 51, 52074 Aachen, Germany; 2 Departamento de Ingeniería Celular y Biocatálisis, Instituto de Biotecnología, UNAM, Universidad Nacional Autónoma de México, Ave. Universidad 2001, Col. Chamilpa, 62210 Cuernavaca, Morelos México; 3 Programa Investigadoras e Investigadores por México del CONACyT, Consejo Nacional de Ciencia y Tecnología, 03940 Mexico City, México

**Keywords:** *A. vinelandii*, Alginate, Yeast extract, Media supplementation, RAMOS, Online monitoring, Micro elements, Amino acids, Biopolymer, Viscosity

## Abstract

**Background:**

In research and production, reproducibility is a key factor, to meet high quality and safety standards and maintain productivity. For microbial fermentations, complex substrates and media components are often used. The complex media components can vary in composition, depending on the lot and manufacturing process. These variations can have an immense impact on the results of biological cultivations. The aim of this work was to investigate and characterize the influence of the complex media component yeast extract on cultivations of *Azotobacter vinelandii* under microaerobic conditions. Under these conditions, the organism produces the biopolymer alginate. The focus of the investigation was on the respiration activity, cell growth and alginate production.

**Results:**

Yeast extracts from 6 different manufacturers and 2 different lots from one manufacturer were evaluated*.* Significant differences on respiratory activity, growth and production were observed. Concentration variations of three different yeast extracts showed that the performance of poorly performing yeast extracts can be improved by simply increasing their concentration. On the other hand, the results with well-performing yeast extracts seem to reach a saturation, when their concentration is increased. Cultivations with poorly performing yeast extract were supplemented with grouped amino acids, single amino acids and micro elements. Beneficial results were obtained with the supplementation of copper sulphate, cysteine or a combination of both. Furthermore, a correlation between the accumulated oxygen transfer and the final viscosity (as a key performance indicator), was established.

**Conclusion:**

The choice of yeast extract is crucial for *A. vinelandii* cultivations, to maintain reproducibility and comparability between cultivations. The proper use of specific yeast extracts allows the cultivation results to be specifically optimised. In addition, supplements can be applied to modify and improve the properties of the alginate. The results only scratch the surface of the underlying mechanisms, as they are not providing explanations on a molecular level. However, the findings show the potential of optimising media containing yeast extract for alginate production with *A. vinelandii,* as well as the potential of targeted supplementation of the media.

**Supplementary Information:**

The online version contains supplementary material available at 10.1186/s12934-023-02112-3.

## Background

*Azotobacter vinelandii* is a gram-negative bacterium that is capable of producing the biopolymer alginate. Alginate consists of two monomers, guluronic acid and mannuronic acid, whose content and distribution can vary [1]. The field of applications is broad. It ranges from its use as an additive in the food industry to water purification for the removal of detrimental substances. Further applications can be found in the medical field, e.g., in wound dressing or drug release systems [2–8]. Most applications are based on the properties of alginate, which can be cross-linked and form gels. Furthermore, alginate forms viscous solutions that have a non-Newtonian, shear-thinning flow behaviour [9].

*A. vinelandii* produces alginate in the process of the formation of cysts, although alginate synthesis is not necessarily followed by the formation of mature cysts. Cyst formation is induced by unfavourable environmental conditions; but this process also occurs naturally during ageing of the culture [10]. As alginate is part of the court layer of growing cysts, it is found extracellularly, and, hence, influences the behaviour of the cultivation medium and leads to an increase in viscosity [11]. Another polymer formed by *A. vinelandii*, is poly-3-hydroxybutyrate (P3HB), mostly present in the inner part of cysts as a storage molecule. *A. vinelandii* distributes its carbon fluxes towards both polymers. Current research aims towards inhibiting or downregulating one pathway to increase the other [12–15].

The productivity of such a biotechnological process depends on the quality of the media components applied. This is particularly true, when the media components are only vaguely defined, such as in the case of complex components or raw materials [16, 17]. This issue has also been addressed by the Food and Drug Administration (FDA). Hence, many industrial biotechnological production facilities closely control and maintain the quality of their complex and raw materials, to guarantee specified yields and quality attributes of their products [18–21].

The characteristics and composition of complex media components are highly dependent on their origin, as well as their manufacturing process [22–24]. This also applies to the complex ingredient yeast extract [25–27]. In addition to the individual process steps, which can influence the end product, one of the major media components used during yeast production also plays a decisive role. For the fermentation of yeast, molasses is often used as a source of carbon, nitrogen and several other nutrients [28–30]. Molasses is a side product of sugar production and, hence, subject to variation [31, 32]. Although producers try to partially compensate for the lack of nutrients in specific molasses, this does not seem to fully compensate for the differences in the nutrient portfolio between different batches of yeast extract [28, 29]. Furthermore, producers state that they use different yeast strains depending on the customer’s desires, which can additionally increase the variation of the final product [33]. The impact of these variations on biotechnological processes has already been reported. Potvin et al. (1997) investigated six different lots of one yeast extract in *Lactobacillus plantarum* cultivations and observed biomass levels varying up to 50% [34]. Cultivating recombinant *Escherichia coli* under lactose-induced conditions, Diederichs et al. (2014) investigated yeast extracts from different manufacturers, as well as different lots from the same supplier. They found that different yeast extracts have a significant impact not only on the respiratory activity of *E. coli*, but also on the volumetric activity of the produced enzymes [35]. Influences on recombinant protein expression with *E. coli* by different yeast extracts have also been reported by Fu et al. (2006) [36]. The impact of complex media components on cultivation results is not only limited to bacteria, but had also been reported for eukaryotes [37, 38].

For the alginate production with *Azotobacter vinelandii*, it has been shown that alginate production is strongly influenced by the choice of the nitrogen source. Different nitrogen sources have been investigated so far in different studies. It was found that complex nitrogen sources, such as peptone or yeast extract, are preferred for higher biomass and alginate yields, as well as higher molecular weights, compared to inorganic nitrogen sources [39–42]. Moreover, Brivonese and Sutherland (1989) stated that the type of the complex nitrogen source peptone is relevant for alginate production [40]. Alginate production is also known to be sensitive towards several factors, such as oxygen supply or shaking and agitation conditions [13, 41, 43, 44]. Several publications have highlighted the influence of media components on growth, alginate production and its characteristics. However, those presented results are at some points controversial, in regards of how certain cultivation conditions influence key cultivation parameters [12, 45]. Different studies have found that the preference for carbon sources can be strain-dependent [40, 46, 47]. As mentioned earlier, the choice of nitrogen source also plays a relevant role in alginate production. The results are less clear about the influence of phosphate. Some studies have shown that, depending on the conditions, both, limiting and sufficiently phosphate-supplied cultivations lead to high alginate concentrations [45, 46].

Other studies revealed a correlation between single-substance supplementations and alginate production. The investigated substances were, among others, MOPS [48, 49], acetate [49] and calcium [50, 51]. A recently published study by Mærk et al. (2020) supplemented, based on metabolic pathway investigations and knowledge of genes involved in regulating alginate synthesis, various nutrients. They found an increase in the alginate concentration induced by the addition of succinate, thiamine and the combination of lysine, methionine and diaminopimelate [52].

For the production of alginate with *A. vinelandii,* a high sensitivity towards different external factors have been reported, as described before [12, 45]. However, to date and the knowledge of the authors, even though different nitrogen sources have been investigated for the growth and alginate production of *A. vinelandii*, no study has been published that investigated different yeast extracts and their influence on alginate production. Hence, this study tries to close this gap, by providing data for several yeast extracts. Furthermore, to enhance productivity and alginate properties, poorly performing yeast extracts were supplemented with specific amino acids, micro elements, trace elements and vitamins.

## Materials and methods

### Strains

For the presented experiments, the wild-type strain *Azotobacter vinelandii* ATCC9046 (American Type Culture Collection, deposited as *A. vinelandii* Lipman, obtained from Cientifica Senna S.A. de C.V, Ciudad de México, Mexico) was used.

### Cultivation media

For all cultivation steps, the modified Burk’s medium was used [53]. All chemicals were purchased, if not stated otherwise, from Carl Roth GmbH & Co. KG (Karlsruhe, Germany), Merck KGaA (Darmstadt, Germany) or Sigma Aldrich Chemie GmbH (Taufkirchen, Germany). For cultivation on agar plates, the medium consists of 0.05 g/L CaSO_4_, 0.027 g/L FeSO_4_ × 7 H_2_O, 0.0029 g/L Na_2_MoO_4_ × 2 H_2_O, 0.2 g/L MgSO_4_ × 7 H_2_O, 0.2 g/L NaCl, 0.66 g/L K_2_HPO_4_, 0.16 g/L KH_2_PO_4_, 1.42 g/L (6.8 mM) 3-(N-morpholino) propane sulfonic acid (MOPS), 20 g/L sucrose and 18 g/L agar. CaSO_4_ was prepared as a separate stock solution (20-fold concentrated) as well as the combination of FeSO_4_ × 7 H_2_O, MgSO_4_ × 7 H_2_O, Na_2_MoO_4_ × 2 H_2_O and NaCl (20-fold concentrated). The rest of the ingredients are combined into a third solution, whose pH is adjusted to 7.2 with 1 M NaOH. The three solutions are then sterilized separately at 121 °C for 21 min. Shortly before cultivation, the solutions are combined under sterile conditions, poured into sterile plates (20 mL per plate) and stored at 4 °C after cooling down.

For liquid preculture, the medium is prepared accordingly to medium for cultivations on plates, without the agar. A yeast extract solution of 150 g/L yeast extract is prepared and sterilized at 121 °C for 21 min. Shortly before cultivation, 3 g/L yeast extract were added to the medium solution. For precultures, the yeast extract Roth Batch 1 was used. Information about all yeast extracts used in this work can be found in Table 1.

**Table 1:** Information on the used yeast extracts for cultivation of *A. vinelandii* ATCC9046

Yeast extract	Abbreviation	Manufacturer	Article name	Article no. & LOT no.
Roth Batch 1	R-1	Carl Roth GmbH + Co. KG., Karslruhe, Germany	Yeast extract, micro grained	2904.5 056239152
Roth Batch 2	R-2	Carl Roth GmbH + Co. KG., Karslruhe, Germany	Yeast extract, pulverized	2363.2 275225976
AppliChem	AC	AppliChem GmbH, Darmstadt, Germany	Yeast extract BioChemica	A1552,1000 6H012930
Oxoid	O	Thermo Fischer Scientific Inc., Waltham, MA, USA	Oxoid^™^ Yeast extract powder	LP0021B 2522291–02
BactoYeast	BY	Thermo Fischer Scientific Inc., Waltham, MA, USA	Gibco^™^ Bacto^™^ Yeast extract	212750 2186295
BioSpringer	BS	Lesaffre & Cie, Marcq-en-Barœul, France	Dry Yeast	0207/0-MG
Merck	M	Merck KGaA, Darmstadt, Germany	Yeast extract, granulated	1.03753.0500 VM957253102

For each yeast extract, the name and abbreviation used in this paper, the manufacturer as well as its article name, article number and lot number, are specified

For liquid main cultures, the medium is prepared as described for liquid precultures, but with a higher MOPS concentration of 14.2 g/L (68 mM). If not stated otherwise, 3 g/L yeast extract of Roth Batch 1 were added.

For supplementation of vitamins, micro elements and trace elements, the respective solutions of SYN6-MES medium, a rich chemically defined medium, developed for cultivations of yeasts, were used [54]. The 100-fold concentrated vitamin solution consists of 0.4 g/L D-biotin and 13.35 g/L thiamine chloride hydrochloride. For the 100-fold concentrated micro element solution, 6.65 g/L (NH_4_)_2_Fe(SO_4_)_2_ × 6 H_2_O, 0.55 g/L CuSO_4_ × 5 H_2_O, 2.0 g/L ZnSO_4_ × 7 H_2_O, 2.65 g/L MnSO_4_ × H_2_O, 6.65 g/L Na_2_EDTA × 2 H_2_O (titration complex III, Carl Roth, Karlsruhe, Germany), were combined. The 100-fold concentrated trace element solution consists of 0.065 mg/L NiSO_4_ × 6 H_2_O, 0.065 mg/L CoCl_2_ × 6 H_2_O, 0.065 mg/L H_3_BO_3_, 0.065 mg/L KI, 0.065 mg/L Na_2_MoO_4_ × 2 H_2_O. All three solutions were sterilized by filtration with a 0.22 µm filter and stored at 4 °C.

For supplementing the ingredients of the micro element solution separately, three solutions were prepared. Each solution consists of either CuSO_4_ × 5 H_2_O, ZnSO_4_ × 7 H_2_O or MnSO_4_ × H_2_O with a concentration of 25 g/L each. The solution was complemented with 6.65 g/L Na_2_EDTA × 2 H_2_O. The solutions were sterilized by filtration with a 0.22 µm filter and stored at 4 °C.

For supplementation of amino acids, solutions based on the work of Müller et al. (2018) were used [55]. The grouping of amino acids was chosen accordingly (see Table 2) and the solutions were prepared as described. The final concentrations for the amino acids in the cultivation medium were: 150 mg/L histidine, 675 mg/L proline, 500 mg/L glutamate, 125 mg/L arginine (group 1: glutamate family), 420 mg/L aspartate, 125 mg/L methionine, 210 mg/L isoleucine, 225 mg/L threonine (group 2: aspartate family), 130 mg/L cysteine, 340 mg/L serine, 175 mg/L glycine (group 3: serine family), 275 mg/L phenylalanine, 250 mg/L tyrosine, 50 mg/L tryptophan (group 4: aromatic family), 240 mg/L alanine, 475 mg/L leucine, 440 mg/L lysine, 325 mg/L valine (group 5: pyruvate family). All solutions were sterilized by filtration with a 0.22 µm filter and stored at 4 °C.

**Table 2:** Groups of amino acids investigated in this work

Group no.	1	2	3	4	5
**Family**	Glutamate	Aspartate	Serine	Aromatic	Pyruvate
**Amino acids**	Histidine	Aspartate	Cysteine	Phenylalanine	Alanine
	Proline	Methionine	Serine	Tyrosine	Leucine
	Glutamate	Isoleucine	Glycine	Tryptophan	Lysine
	Arginine	Threonine			Valine

### Microbial cultivation

All liquid cultivations were conducted using the Kuhner TOM device (Kuhner shaker GmbH, Herzogenrath, Germany) or the comparable in-house build prototype based on the RAMOS technology [56, 57]. Both devices monitor the respiratory activity of microbial cultivations in eight parallel specially designed shake flasks with a screw cap. This allows to equip the shake flasks with a special lid that is connected to an aeration system with a gas analysis. This includes oxygen as well as carbon dioxide sensors. The aeration rate is chosen to mimic the gas conditions in commonly used standard shake flasks with cotton or paper plugs, to maintain comparability. The specially designed shake flasks differ in the upper part to standard shake flasks, due to the screw cap. However, the geometry of the lower part of the flasks, which is in contact with the rotating culture liquid, is identical and assures comparability of cultures between standard shake flasks and RAMOS shake flasks. It has been proven in multiple investigations that the results from ordinary shake flasks and from modified shake flasks used for the RAMOS experiment are identical [58–61]. Both devices (Kuhner Tom and RAMOS) provide online signals of the oxygen (OTR) and the carbon dioxide (CTR) transfer rate. This allows the calculation of the accumulated oxygen transfer (AOT), which equals the integral of the OTR. For liquid precultures, agar plates were inoculated with 200 µL of a cryo stock (stored at − 80 °C) and incubated for approximately 72 h at 30 °C. With an inoculation loop, biomass was transferred to a 1.5 mL reaction tube and suspended in 1 mL of preculture medium by pipetting. The inoculated medium was then transferred to the rest of the medium batch and then distributed to the flasks. Cultivation was conducted in two 250 mL RAMOS-flasks with a filling volume of 50 mL each. The preculture flasks were shaken at 29 °C for approximately 24 h at a shaking frequency of 155 rpm and a shaking diameter of 50 mm. Conditions were chosen according to Gómez-Pazarin et al. (2016) for precultures and García et al. (2020) for main cultures [53, 62]. To assure comparability to the before mentioned references, while using different shaking diameters, flask sizes and filling volumes, the maximum oxygen transfer capacity (OTR_max_), according to the equation developed by Meier et al. (2016), was calculated for the respective conditions [63]. Gómez-Pazarin et al. (2016) cultivated the preculture at 29 °C in 500 mL flasks with a filling volume of 100 mL at a shaking frequency of 200 rpm and a shaking diameter of 25 mm, which equals a theoretical OTR_max_ of 9.3 mmol/L/h. García et al. (2020) cultivated the main culture at 29 °C in 250 mL flasks with a filling volume of 50 mL at a shaking frequency of 200 rpm and a shaking diameter of 25 mm, which equals a theoretical OTR_max_ of 10.0 mmol/L/h. With the aim to reach a comparable OTR_max_ as with the conditions by Gómez-Pazarin et al. (2016) and García et al. (2020), the shaking frequency was then adjusted for the experiments presented in this paper. Hence, with the presented conditions, the theoretical OTR_max_ for the precultures in this work is 9.3 mmol/L/h and for the main cultures 10.0 mmol/L/h.

To inoculate the main culture with 10 vol-% of preculture, the preculture was centrifuged in sterile 50 mL falcon tubes at 4000 rpm and 4 °C for 10 min. The supernatant was discarded and the remaining cell pellet was suspended in the main culture medium. Main cultivations were carried out with 250 mL RAMOS-flasks and a filling volume of 50 mL. The flasks were shaken at 29 °C at a shaking frequency of 165 rpm and a shaking diameter of 50 mm for approximately 72 h. All experiments were conducted with saturated moist air to minimize evaporation of the culture medium during cultivation.

For sampling during cultivation, parallel standard shake flasks were prepared as mentioned above and incubated at comparable conditions in a shaking hood with 85% air humidity.

### Analytical methods

The pH of the freshly inoculated medium, as well as the pH at the end of cultivation was measured at room temperature with a pH/ORP meter (HI 2211, Hanna Instruments Inc., Smithfield, RI, USA) and an InLab Solids pH electrode (Mettler Toledo International Inc, Columbus, OH, USA).

At the end of cultivation, the cell dry weight was determined gravimetrically in quadruplicates, based on a method described earlier [44]. Per replicate, 1 mL of culture broth was mixed with 100 µL 0.1 M EDTA and 100 µL 1 M NaCl in a 2 mL reaction tube, whose empty weight has been determined beforehand. The tube was then centrifuged for 15 min at 14,000 rpm and then dried at 80 °C for at least 24 h. After cooling down in a desiccator for one hour, the weight was determined with an analytical scale.

Alginate dry weight was determined at the end of cultivation in triplicates, based on a method described earlier [64]. Per replicate, 10 mL of culture broth was mixed with 1 mL 0.1 M EDTA and 1 mL 1 M NaCl in a 15 mL falcon tube. The tube was then centrifuged for 20 min at 10,000 rpm. The supernatant was transferred to a 50 mL falcon tube. For precipitation, 30 mL of 2-propanol were added to the supernatant and incubated for 15 min on ice. The tubes were then centrifuged for 15 min at 4000 rpm. The liquid phase was discarded and the alginate was transferred to weighed 5 mL reaction tubes and dried at 80 °C for at least 24 h. After cooling down for an hour in a desiccator, the weight of the dried alginate was determined using an analytical scale.

To determine the molecular weight and the acetylation degree of the alginate, a solution of 20 g/L dried alginate in demineralized water was prepared. For molecular weight measurements, this solution was diluted to 5 g/L and then filtered using a 0.45 µm membrane made of regenerated cellulose (RC). The samples were analysed via gel permeation chromatography (GPC, EcoSec HLC-8320GPC, Tosoh Bioscience GmbH, Griesheim, Germany), as previously described [62]. A combination of three columns was used (Ultrahydrogel Guard column 6 × 40 mm, WAT011565; Ultrahydrogel Linear 7.8 × 300 mm, WAT011545; Ultrahydrogel 500 7.8 × 300 mm, WAT011530, Waters GmbH, Eschborn, Germany). A mobile phase of 0.1 M NaNO_3_ was applied with a flow rate of 0.9 mL/min at a temperature of 40 °C. Per sample, 100 µL were injected. For molecular weight determination, polyethylene glycol (PEG) and polyethylene oxide (PEO) standards with molecular weights between 238 and 1,300,000 Da (ReadyCal-Kit PEO/PEG, PSS-peokitr1 and poly(ethylene oxide), 1,300,000 Da, PSS-peo1.3 m, PSS GmbH, Mainz, Germany) were used.

For the determination of the acetylation degree, an acetylation assay was performed as previously described [13]. 500 µL of the 20 g/L alginate solution was mixed with 500 µL of 1 M NaOH and incubated for 2 h at 80 °C in a ThermoMixer^™^ (Thermo Fischer Scientific GmbH, Dreieich, Germany). Then, 625 µL 1.5 M H_3_PO_4_ solution were added, mixed and centrifuged for 15 min at 11,000 rpm. The aqueous phase was then filtered through a 0.22 µm cellulose acetate membrane. The acetate content of the samples was determined using a HPLC (Shimadzu Europa GmbH, Duisburg, Germany) with an organic acid column (ROA-Organic Acid H + , Phenomenex Inc., Germany). The measurement was performed with a mobile phase of 5 mM H_2_SO_4_, a flow rate of 0.8 mL/min and at a temperature of 60 °C. The acetylation degree is the weight ratio of acetate to alginate (after dry-weight measurement) per sample.

The viscosity of the culture broth was measured within 6 h after the end of the RAMOS cultivation, which usually lasts 72 h. Viscosity measurements were performed using a rheometer (MCR 301, Anton Paar GmbH, Graz, Austria) with a cone-plate measuring system (CP50-0.5/TG). The viscosity was measured with a gap of 0.054 mm between cone and plate, at shear rates from 100 to 5000 1/s and a temperature of 22 °C. Final broth viscosities are reported as those evaluated at shear rates of 316 1/s. This value is in the range of shear rates, reached in shake flasks applying the described conditions, and is often used in literature to report results for this microbial system and, hence, allows for comparisons [53, 62, 65].

Sucrose concentration was determined using the 3,5-Dinitrosalicylic acid (DNS) assay [66]. Each sample was centrifuged at 14,000 rpm for 15 min. A solution of 2.5 g/L of invertase (Fluka BioChemika, Honeywell Inc., Charlotte, NC, USA) in citrate buffer (0.35 M, pH 4.6) was prepared. To convert sucrose into glucose and fructose, 10 µL of the invertase solution was added to 90 µL of sample in a 1.5 mL reaction tube and incubated at room temperature for 10 min. Then, 100 µL DNS solution (16 g/L NaOH, 300 g/L potassium sodium tartrate, 10 g/L DNS) were added. The mixture was incubated at 100 °C for 5 min using the ThermoMixer^™^ and then cooled down for 15 min on ice. To each sample, 1 mL of demineralized water was added. To determine concentrations, standard solutions consisting of sucrose of known concentrations were prepared accordingly to cultivation samples. Each sample was measured in triplicates by adding 120 µL per replicate into wells of a transparent, flat bottom 96-well plate. Absorbance was measured in a plate reader (Synergy 4, BioTek, Winooski, VT, USA) at 540 nm.

### Standard deviation and statistical analysis

For this work, standard deviations were calculated, if at least three replicates were available. The experimental design required that certain yeast extracts were used more often in cultivations. They served as reference and enabled to monitor reproducibility over the course of all experiments. This is especially the case for Roth yeast extracts and, to some extent, for AppliChem yeast extracts. Hence, the number of replicates is different.

The error bars shown are sometimes larger than the observed effects. This may be partly due to the fact that the handling of some of the viscous and gel-like solutions was challenging. Higher viscosities might be another reason for a higher standard deviation. The cells form more aggregates, which might not only limit cell mobility, but also the exchange of nutrients and gases [67]. The aggregates lead to more inhomogeneities within the culture broth, which could lead to higher deviations.

In addition, statistical tests were carried out to evaluate the presented data. For this, the free software RStudio^®^ (Posit Software PBC, formerly RStudio PBC, Boston, Massachusetts, USA, version 2022.12.0 + 353) was used. For the presented data, a one-factorial ANOVA was used for statistical analysis followed by a paired t-test with adjustment of the p-value based on Bonferroni. A p-value < 0.05 was considered significant.

## Results and discussion

### Cultivation of *A. vinelandii* under microaerobic conditions

**Fig. 1:**
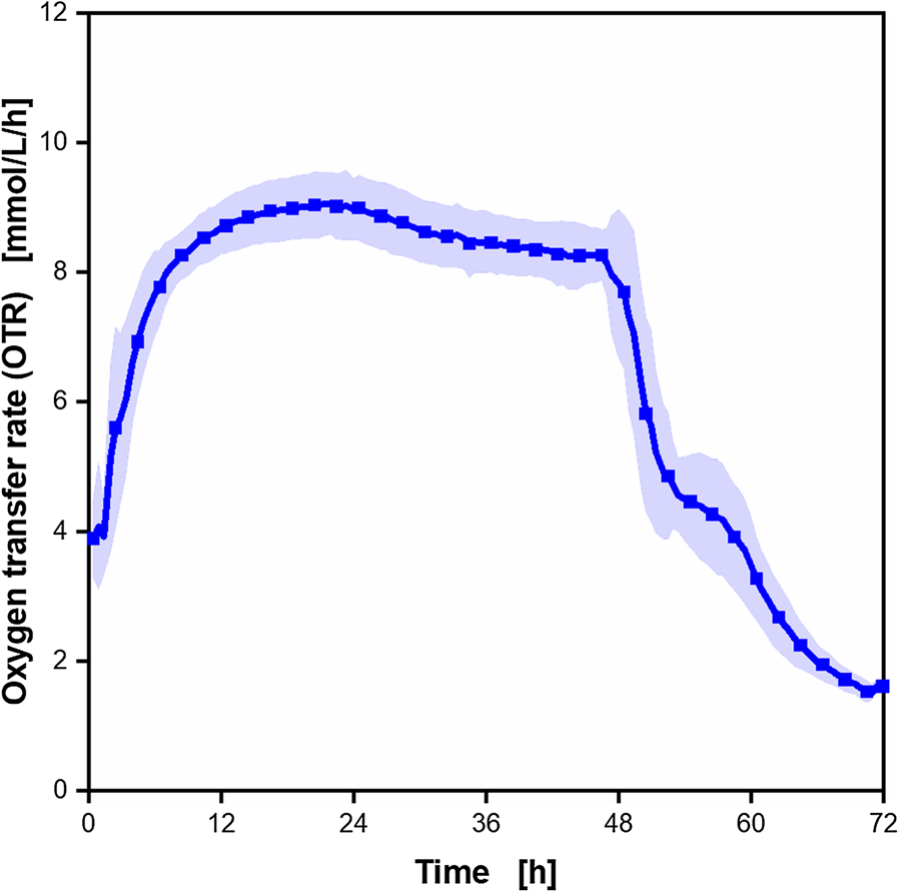
**Oxygen transfer rate of microaerobic** ***A. vinelandii* ATCC9046 cultivations**. Cultivations were conducted in modified Burk’s medium with 3 g/L yeast extract (from Roth, Batch 1). The depicted OTR is the mean of 18 replicates from 12 different experiments, conducted over the course of 13 months. The corresponding standard deviation is depicted as a shadow. For clarity, only every fifth measuring point is shown. The OTR curves of each replicate are depicted in Additional file 1: Fig. S1A. Cultivation conditions: 250 mL shake flasks, initial pH = 7.2, filling volume V_L_ = 50 mL, temperature T = 29 °C, shaking frequency n = 165 rpm, shaking diameter d_0_ = 50 mm

It is known that the cultivation of *A. vinelandii* ATCC 9046 under microaerobic (oxygen-limited) conditions, promotes the production of alginate, especially alginates with a high molecular weight [44, 53, 64, 68, 69]. Therefore, firstly, an *A. vinelandii* reference cultivation under microaerobic conditions using modified Burk’s medium was conducted as a basis for further investigations. The mean oxygen transfer rate (OTR) from 18 RAMOS cultivations from 12 independent experiments, conducted under the same conditions over a course of 13 months, were calculated. For each experiment, 8 flasks were available and each condition was tested in biological duplicates. Accordingly, three conditions per experiment could be tested in addition to the reference. In a few experiments, no data could be recorded for one of the biological replicates, due to sensor failure (e.g., because of limited service life). For such experiments, online data are only available for one biological replicate and not, as usual, for two. Therefore, not twice as many OTR data of cultivations as experiments are available. The results are shown in Fig. 1.

The curve of the OTR shows the typical plateau shape of a microaerobic cultivation (oxygen limitation) [56]. The amount of the provided oxygen per time is limited by the chosen cultivation conditions (relatively high filling volume and low shaking frequency). As the cultivation conditions are kept constant, a constant OTR is observed during this period of oxygen limitation. The maximum OTR of 9 mmol/L/h is reached after 18 h. After 46 h, the curve declines and forms a shoulder at 54 h. After 66 h, an OTR below 2 mmol/L/h is achieved, which is associated with very low metabolic activity. No further changes in the OTR could be observed for similar experiments with a longer cultivation time (data not shown). Hence, the RAMOS experiments were stopped from this experiment on after 72 h. The standard deviations are low and, hence, a good reproducibility can be assumed. All 18 single cultivations are displayed in Additional file 1: Fig. S1A. Similar shapes of OTR curves have been described before for cultivations in shake flasks [62, 70, 71]. Only minor differences between the present study and previously published papers, especially in the reached maximum OTR as well as in the formation of the shoulder, could be observed. Those differences are almost certainly the results of minor variations in the cultivation conditions such as medium composition, used strains and shaking parameters.

**Fig. 2:**
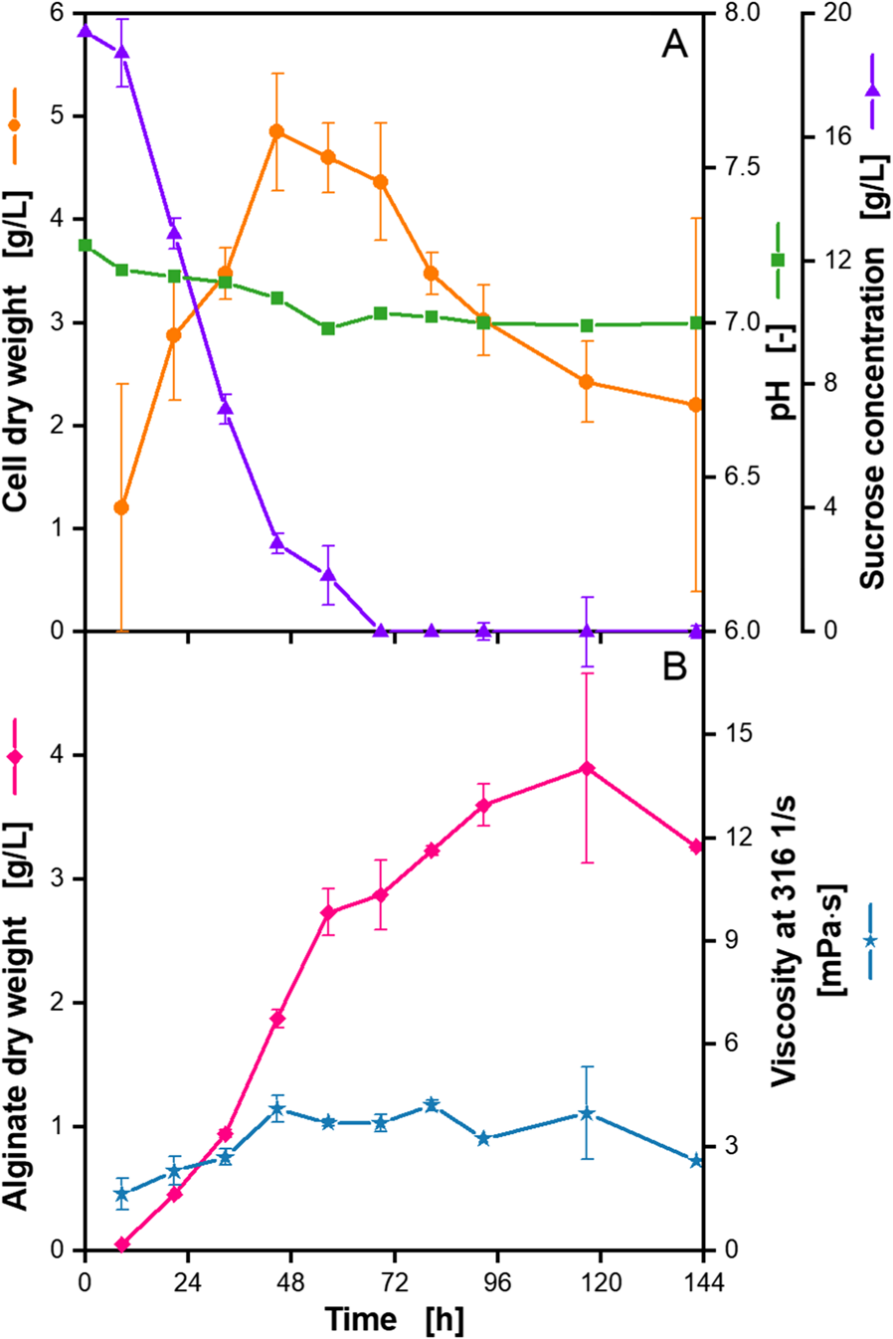
**Analysis of offline samples of a microaerobic** ***A. vinelandii* ATCC9046 cultivation**. Cultivations were conducted in parallel shake flasks in modified Burk’s medium with 3 g/L yeast extract (from Roth, Batch 1). For each sample point, one new flask was removed from the shaker for analysis and discarded afterwards. Standard deviation is given for n ≥ 3 **A**: Cell dry weight, n = 4. The standard deviation for the sucrose concentration is given for each sample of the assay that was measured three times. **B**: Alginate dry weight, n = 3; broth viscosity, n = 4, except for time points 92 h and 142 h, which were determined in duplicates. Cultivation conditions: 250 mL shake flasks, initial pH = 7.2, filling volume V_L_ = 50 mL, temperature T = 29 °C, shaking frequency n = 165 rpm, shaking diameter d_0_ = 50 mm

To monitor the evolution of certain key cultivation parameters, including cell dry weight, pH and alginate concentration, a cultivation in parallel shake flasks was conducted, using the same conditions as for the above-described RAMOS experiment. For each sample point, one sample flask was removed from the shaker, utilized for analysis and discarded afterwards. The shake flask experiments were conducted for a longer cultivation time than the parallel RAMOS experiment to investigate, whether key cultivation parameters change during the phase with no respiration activity. The results are presented in Fig. 2.

During the growth phase, which approximately equals the time span of the first 48 h of cultivation, an increase in cell dry weight is observed. At the same time, a decrease in the concentration of the main carbon source sucrose occurred (Fig. 2A). Sucrose is depleted after 68 h, which equals roughly the time point, where the OTR is at its lowest level and, hence, the metabolic active phase is over. After 48 h, a decrease in cell dry weight is visible, which might be due to cells dying and rupturing, as probably many nutrients, including sucrose, are depleted [72, 73]. Furthermore, morphological changes due to stressful situations, such as nutrient depletion, may contribute to the observed biomass data [74]. The initial pH decreases from 7.3 to around 7.0 during the growth phase. No further changes in pH are visible after the end of the metabolic active phase. The used amount of 68 mM MOPS buffer, which is 10 times higher than the standard concentration of 6.8 mM MOPS buffer, stabilized the pH. This is in accordance with a study by Clementi et al. (1995). They used 50 mM MOPS buffer in a different medium and confirmed that it stabilized the pH between 7.5 and 6.5 [49]. Peña et al. (1997 & 2006) reported of cultivations of the same strain in the same medium with 6.8 mM MOPS buffer. At the end of the cultivation after 72 h, a pH of 5.55 was reached [48, 64]. Their cultivation conditions result in an OTR_max_ calculated after Meier et al. (2016), which is similar to the OTR_max_ in this study and allows to compare the results [63].

Alginate dry weight, shown in Fig. 2B, increases beyond the growth phase, confirming that product formation is only partially growth associated. This is in accordance with the literature [64, 70]. After 120 h, a maximum alginate dry weight of 3.9 g/L is reached. The amount of alginate produced is comparable to findings reported in the literature for similar conditions with the same strain in shake flasks [39, 62]. Alginate production seems to have reached a maximum after 96 h. This is interesting, since the carbon source sucrose is already used up after 68 h. *A. vinelandii* produces poly-3-hydroxybutyrate as an internal storage molecule, which might then be used as an alternative carbon source for alginate production [75, 76]. After 96 h, no further alginate production can be observed. This corresponds to the fact that the cells are less or even completely inactive, as discussed above.

The viscosity of the culture broth increases up to 4.1 mPa·s after 48 h. No further increase in viscosity is visible, despite the rising alginate concentration. It is known that the alginate concentration is not the only factor affecting the viscosity of a solution. The molecular properties of the alginate, such as mean molecular weight, the acetylation degree and the content of the monomers, play a key role [9, 45, 48, 77]. For the present experiment, this cannot be conclusively clarified with certainty, as neither molecular weight nor degree of acetylation were measured. But it is possible that lyases secreted by *A. vinelandii* degrade the produced alginate into shorter chains and, thus, counteract the potential viscosity increase due to the rising alginate concentration [67, 68, 78].

To conclude, a cultivation time of 72 h is appropriate to monitor the metabolic active phase of *A. vinelandii*, in regards of the key cultivation parameters, biomass and alginate production.

### The choice of yeast extract influences the cultivation of *A. vinelandii* under microaerobic conditions

**Fig. 3:**
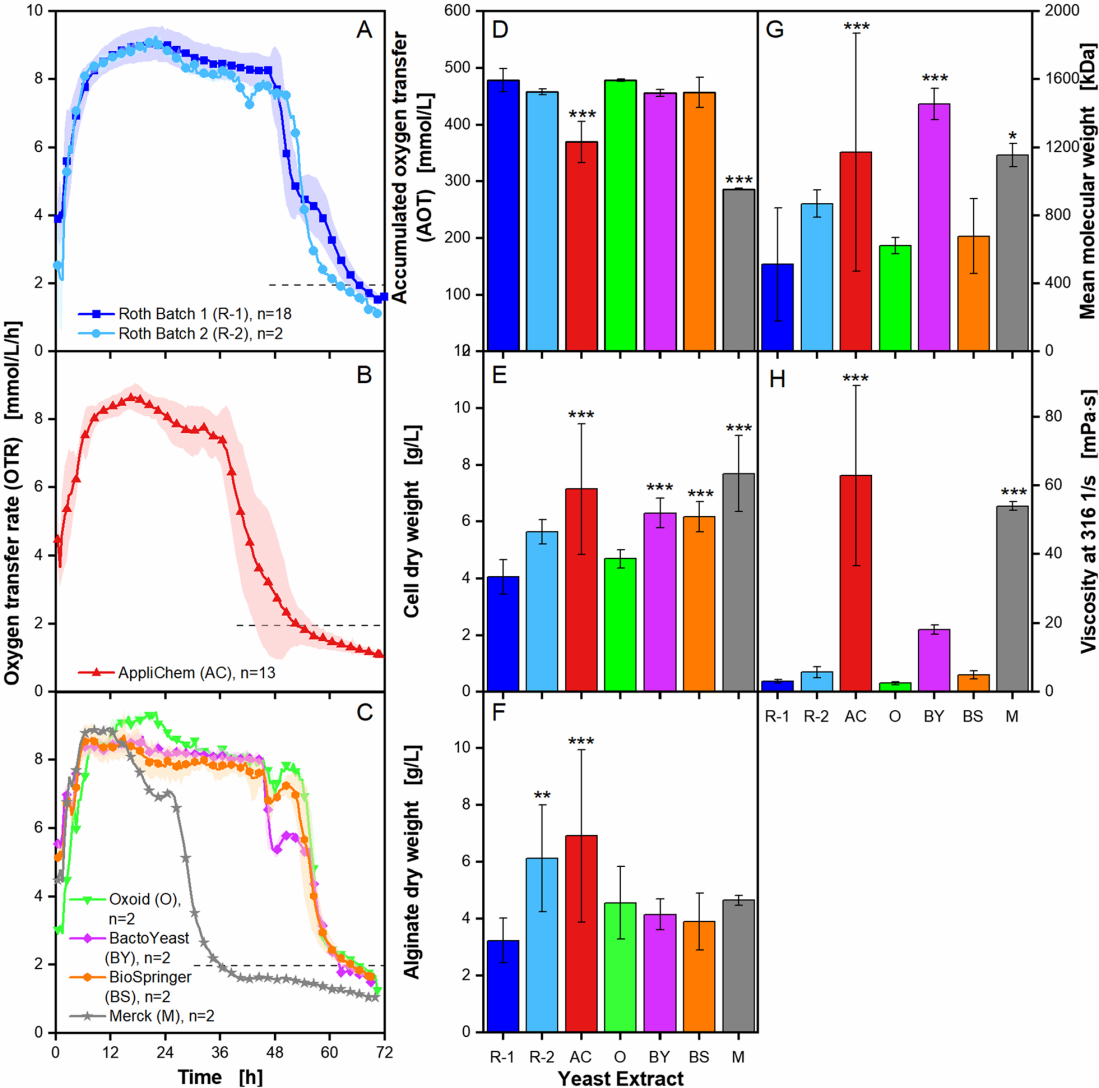
**Effect of different yeast extracts on microaerobic** ***A. vinelandii* ATCC9046 cultivations***.* Cultivations were conducted in modified Burk’s medium with 3 g/L yeast extract from different manufacturers. Standard deviations are given for n ≥ 3, represented by a shadow or error bars and are in some cases so small that they are not well recognizable. Significant differences to the reference (Roth Batch 1) are marked with asterisks, corresponding to the significance level (* for 0.01 < p < 0.05, ** for 0.001 < p < 0.01 and *** for p < 0.001). **A**–**D**: For clarity, only every fifth measuring point of the OTR curves is shown. The number of replicates is given with n; if n = 2, the mean value is shown and the shadow depicts the difference between the two replicates. The OTR curves for each replicate of cultivations with Roth Batch 1 and AppliChem are shown in Additional file 1: Fig. S1A, B, respectively. The horizontal dashed line depicts the OTR value of 2 mmol/L/h for an easier comparison of the nominal cultivation time. **E**–**H**: All given values are mean values, calculated from the following number of replicates: cell dry weight, n ≥ 8; alginate dry weight, n ≥ 6; mean molecular weight, n ≥ 5; broth viscosity, n ≥ 4. The flow curves and the acetylation degree are provided in Additional file 1: Fig. S3. Cultivation conditions: 250 mL shake flasks, initial pH = 7.2, filling volume V_L_ = 50 mL, temperature T = 29 °C, shaking frequency n = 165 rpm, shaking diameter d_0_ = 50 mm

The influence of different yeast extracts on the metabolic activity of *A. vinelandii* and its alginate production was investigated by using two different lots from one manufacturer, as well as yeast extracts from six different manufacturers. The details of the applied yeast extracts are listed in Table 1. The results of these cultivations are depicted in Fig. 3.

The reference cultivation from Fig. 1 has been conducted with yeast extract from the manufacturer Roth and is shown in dark blue in Fig. 3A. From the same manufacturer, a second lot was examined and is depicted in the same diagram in light blue. The primary distinction between the two cultivations is the shoulder formation. For Roth Batch 2, the shoulder in the OTR is shown at 42 h, 12 h earlier compared to the reference cultivation. The OTR of the cultivation with Roth Batch 2 also decreased only slightly from the plateau to the shoulder to around 7.8 mmol/L/h, whereas for the reference cultivation, the shoulder forms at an OTR of 4.4 mmol/L/h. Cultivations with Roth Batch 2 reach 1.9 times higher alginate dry weights than cultivations with yeast extract Roth Batch 1 (Fig. 3F). No further significant differences to the reference could be detected for the other investigated parameters.

To further investigate the influence of different yeast extracts, *A. vinelandii* cultivations with yeast extracts from five other manufacturers have been conducted and compared to the reference cultivation. A major difference in the OTR is the formation of the shoulder in later stages of the cultivation (Fig. 3A–C). Cultivations with yeast extracts by Oxoid, BactoYeast and BioSpringer show a shoulder after 48 h of cultivation, but at different OTR levels (Fig. 3C). For comparison, the end of cultivation is referred to as the time point, the OTR drops to levels lower than 2 mmol/L/h. This low OTR is associated with low to no metabolic activity. Hence, the reference cultivation ends after 66 h (Fig. 3A). Cultivations conducted with yeast extract by AppliChem end at approx. 52 h and cultivations with yeast extract by Merck terminate at 36 h (Fig. 3A–C). This discrepancy in cultivation time is also reflected in the accumulated oxygen transfer (AOT), which is up to 1.7 times lower for those respective shorter cultivations (Fig. 3D, cultivations with AppliChem and Merck yeast extracts). The cell dry weight is up to 1.9 times higher for cultivations using AppliChem, BactoYeast, Biospringer and Merck yeast extracts than for the reference cultivation (Fig. 3E). A significant influence on alginate production is only visible for cultivations with AppliChem yeast extract, leading to values twice as high as the reference (Fig. 3F). The mean molecular weight increases up to 2.8 times for cultivations using BactoYeast, Merck and AppliChem yeast extracts (Fig. 3G).

No significant effects of different extracts on the acetylation degree were found. (Additional file 1: Fig. S3A). In contrast to that, the effect of yeast extracts on the culture broth viscosity is rather large (Fig. 3H). Cultivations with Roth Batch 1 only reach viscosities around 3 mPa·s, whereas the cultivations with AppliChem and Merck reach considerably higher viscosities, of around 50 to 60 mPa·s. The viscosity was measured as a function of the shear rate and the respective flow curves with the typical shear-thinning behaviour for alginate solutions can be seen in Additional file 1: Fig. S3B [9, 10].

The obtained results highlight that the choice of yeast extract strongly impacts the performance of a cultivation. These findings are in accordance to publications that investigated the influence of yeast extracts with other microbial systems [22, 35, 37].

Interestingly, neither the respiratory activity nor growth and alginate production are affected, when *A. vinelandii* is cultivated at sufficient oxygen supply (non-limited conditions) with different yeast extracts (Additional file 1: Fig. S2). Differences in the culture performance only appear under oxygen limitation. It can be assumed that the influence of the composition of the yeast extract is related to alginate production, which is enhanced under microaerobic conditions [44, 53, 68].

The main variations observed in the OTR of *A. vinelandii* cultivations with different yeast extracts were variations in the shoulder formation, as well as the time of the metabolic active phase. A shoulder formation or double peak formation in the OTR usually refers to a switch in metabolism, for example during diauxic growth, when the organism changes from one carbon source to another [60, 79, 80]. The shoulder formation in the presented experiments may also originate from such a metabolic switch. García et al. (2020) proposed that the shoulder of *A. vinelandii* cultivations is connected to the amount of nitrogen provided through the medium [62]. Therefore, a metabolic switch from non-diazotrophic metabolism to diazotrophic metabolism is imaginable. So far, the origin of this shoulder has not been determined. Likewise, a verified explanation is still missing for the correlation between the time frame of high metabolic activity and the accumulated oxygen transfer (AOT). Usually, a lower AOT is associated with lower metabolic activity, resulting in reduced cell growth and/or production of a target product. Increasing initial carbon source concentrations has been shown to usually lead to higher AOTs and to an increase in cell growth and/or productivity [80–82]. Decreases in the AOT are often associated with less favourable conditions or metabolic burdens and, hence, with a decline of growth and production [83, 84]. Thus, the results obtained in this study were unexpected, with a lower AOT being clearly associated with higher growth and higher broth viscosity.

### Investigating the influence of concentration variations of three different yeast extracts on *A. vinelandii* cultivations

**Fig. 4:**
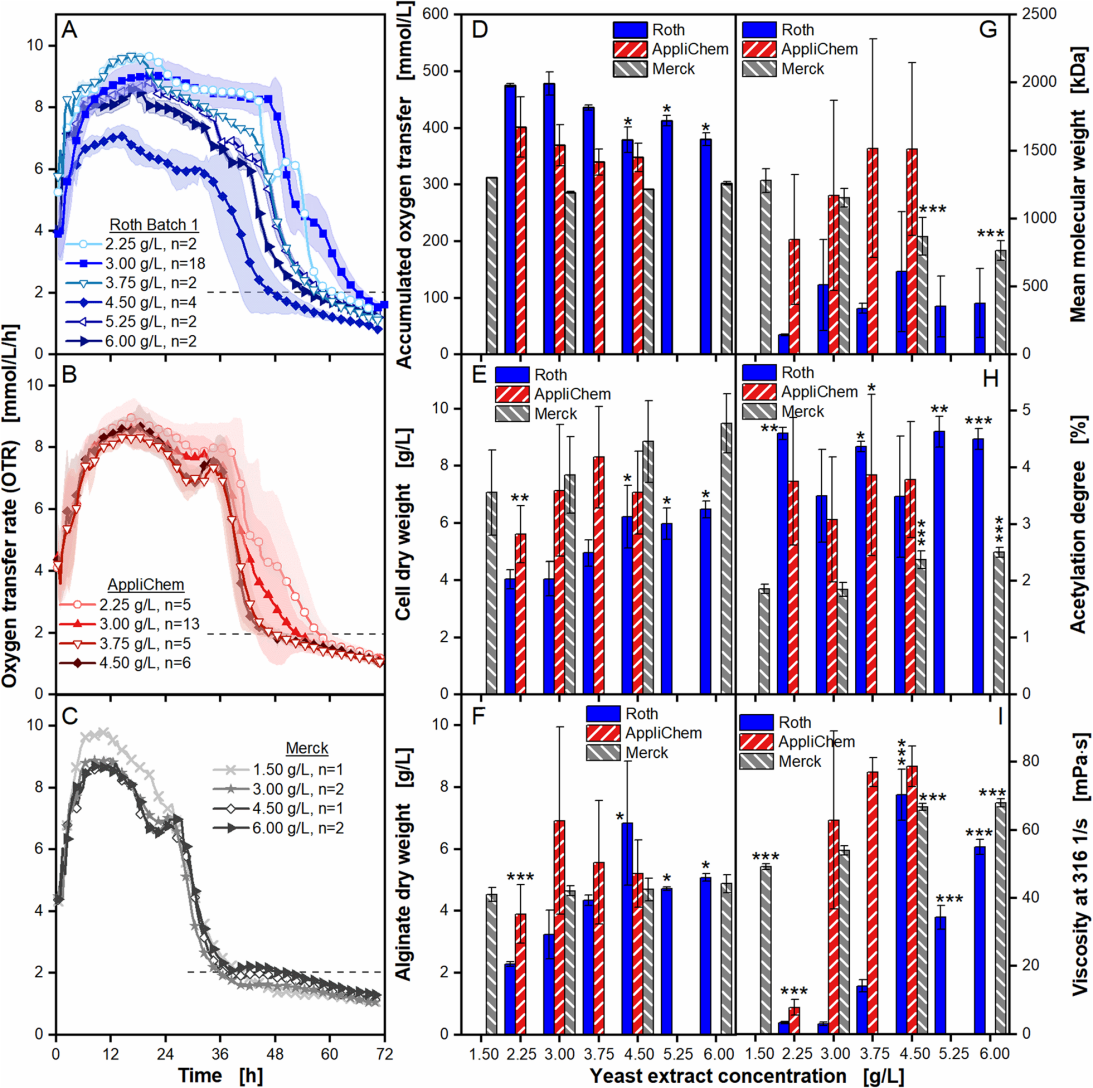
**Effect of different yeast extract concentrations on microaerobic** ***A. vinelandii* ATCC9046 cultivations**. Cultivations were conducted in modified Burk’s medium with different yeast extract concentrations from three different manufacturers (Roth, AppliChem and Merck). Standard deviation was calculated for n ≥ 3. Significant differences to the reference (3 g/L yeast extract) are marked with asterisks, corresponding to the significance level (* for 0.01 < p < 0.05, ** for 0.001 < p < 0.01 and *** for p < 0.001). **A**–**D**: For clarity, only every fifth measuring point of the OTR curves is shown. The number of replicates is given with n; if n = 2, the mean value is shown and the shadow depicts the difference of the two replicates. The horizontal dashed line depicts the OTR value of 2 mmol/L/h for an easier comparison of the nominal cultivation time. **E**–**I**: All values are given as mean values, calculated from the following number of replicates: cell dry weight, n ≥ 8; alginate dry weight, n ≥ 6; mean molecular weight and acetylation degree, n ≥ 6; broth viscosity, n ≥ 4. The flow curves are provided in Additional file 1: Fig S4. Cultivation conditions: 250 mL shake flasks, initial pH = 7.2, filling volume V_L_ = 50 mL, temperature T = 29 °C, shaking frequency n = 165 rpm, shaking diameter d_0_ = 50 mm

As previously shown, the yeast extracts have an impact on cultivations of *A. vinelandii,* exhibiting different unique respiratory activities as well as alginate production. Since all other cultivation parameters have been kept constant, it can be stated that the observed variations result from differences in the yeast extract composition. A concentration increase of a yeast extract might positively influence the performance of *A. vinelandii,* with regard to growth and production under microaerobic conditions. Therefore, three yeast extracts from Roth Batch 1, AppliChem and Merck were chosen for further investigation. These yeast extracts showed a noticeable increase in final broth viscosity and led to overall better results, compared to cultivations with the reference yeast extract Roth Batch 1 (Fig. 3). For each yeast extract, the standard concentration of 3 g/L was increased up to 4.5 g/L and 6 g/L. Additionally, one lower concentration was investigated. For the poorer performing yeast extract from Roth, more steps in between these concentrations were added for a more detailed analysis of how the concentration variation affects the cultivation. The results are shown in Fig. 4.

Cultivations with Roth Batch 1 are presented in Fig. 4A. It can be observed, that an increase in yeast extract concentration leads to an earlier decrease of the OTR, resulting in a up to 18 h shorter metabolic active phase. Furthermore, the concentration variation of the yeast extract leads to variations in the shoulder formation, but no clear trends can be identified. The evolution of the OTR does not change in a linear manner in regards to the concentration increase. Currently, no explanation can be given for this observation and further experiments are required. For cultivations with AppliChem yeast extract (Fig. 4B) and Merck yeast extract (Fig. 4C), the concentration variation showed no influence on respiratory activity and the AOT (Fig. 4D). In contrast to that, the AOT has a decreasing trend with increasing concentration of Roth Batch 1 yeast extract, starting at a concentration of 4.5 g/L. The cultivation with Roth Batch 1 is also the only one showing an increase in cell dry weight and alginate concentration with increasing yeast extract concentration (Fig. 4E, F). Only for AppliChem cultivations with 2.25 g/L yeast extract, a significant decrease of cell dry weight and alginate dry weight could be detected compared to the reference. The only yeast extract showing a concentration influence on the mean molecular weight was the yeast extract by Merck. An enhanced concentration of 5.25 g/L or more leads to a decrease in mean molecular weight (Fig. 4G). Interestingly, this is correlated to an increase in acetylation degree and in viscosity (Fig. 4H, I). The increase in viscosity despite a lower mean molecular weight and a constant concentration (Fig. 4F) might be explained by molecular properties, such as monomer content and sequence in the macromolecule, that have not been investigated in this work [85–87]. For AppliChem yeast extract cultivations, only the concentration of 3.75 g/L shows a significant increase in the acetylation degree to the reference. There seems to be no correlation with any other investigated parameter. The increased concentration of Roth Batch 1 leads further to an increase in broth viscosity (Fig. 4I). The concentration of 2.25 g/L AppliChem yeast extract leads to a lower viscosity than the reference, which might originate from the overall lower alginate production. The flow curves from the viscosity measurements are given in Additional file 1: Fig. S3. They correspond well to the already discussed results.

Future research should include the analysis of all molecular properties of the produced alginate, enhancing the understanding of the impact of the nutrients. Though, it has to be noted that the presence of certain ions in the culture broth could also influence the viscosity, as the ions might interact with the produced alginate [1, 5, 88]. Depending on the composition of the yeast extracts, different ions in varying amounts might be present, influencing the viscosity differently. Based on the available data, it is quite difficult to differentiate, which influence on the viscosity dominates, whether it is the molecular properties of the alginate or the present ions. In the end, if the molecular properties change, the impact on the viscosity will change.

It can be concluded that depending on the yeast extract, an increase in yeast extract concentration can lead to an improvement in growth and production. Which parameters are influenced to what extent, depends on the type of yeast extract. Furthermore, it seems that a saturation is reached at certain yeast extract concentrations that stops further improvement of growth and alginate production under microaerobic conditions. Probably the cultivation lacks nutrients that are not provided by the yeast extracts. The results also show that it is possible to a certain extent to compensate the poor performance of a yeast extract, such as Roth Batch 1, by increasing its concentration. Thereby, a similar performance can be obtained as with the high performing yeast extracts.

By changing the yeast extract concentration and keeping the sucrose concentration constant, the C/N ratio varies. The higher the yeast extract concentration, the lower the C/N ratio. Literature reports that the C/N ratio is associated with changes in growth and alginate production. For example, Zapata-Vélez et al. [39] investigated different nitrogen sources and varied the C/N ratio with peptone, which is a complex nitrogen source, similar to yeast extract [39]. They reported that lower C/N ratios down to 14.4/1, by using sucrose and peptone, led to higher alginate and biomass concentrations. This is in accordance with some of the results presented in this study. The exact composition of the yeast extracts used in this study is not available, so the values given in Zapata-Vélez’ work are used instead. Based on the composition of yeast extract estimated by Zapata-Vélez et al. [39] (10.90% nitrogen and 36.94% carbon), the lowest C/N ratio with 20 g/L sucrose and 6 g/L yeast extract in this work is 16.3. Another study by Cho et al. [89] reported similar results [89]. They investigated the C/N ratio based on swine wastewater supplemented with glucose and ammonium chloride and cultivated the mutant strain UDW of *A. vinelandii* ATCC 53,799. They reported higher cell dry weights for lower C/N ratios, achieving the highest cell dry weights for ratios of 22/1 and 17/1. Nonetheless, for cultivations with AppliChem and Merck, no increase with decreasing C/N ratio could be observed. Therefore, the results obtained in this work are to some extent comparable to literature findings for *A. vinelandii*. Additionally, variations of production performance for different manufacturers of yeast extract are emphasized. Zapata-Vélez et al. [39] only investigated complex nitrogen sources, including peptone and yeast extract, from one manufacturer (BD-Difco), which might explain the differences in the results as discussed above [39].

### Supplementation of vitamins, micro elements and trace elements to *A. vinelandii* cultivations under microaerobic conditions

**Fig. 5:**
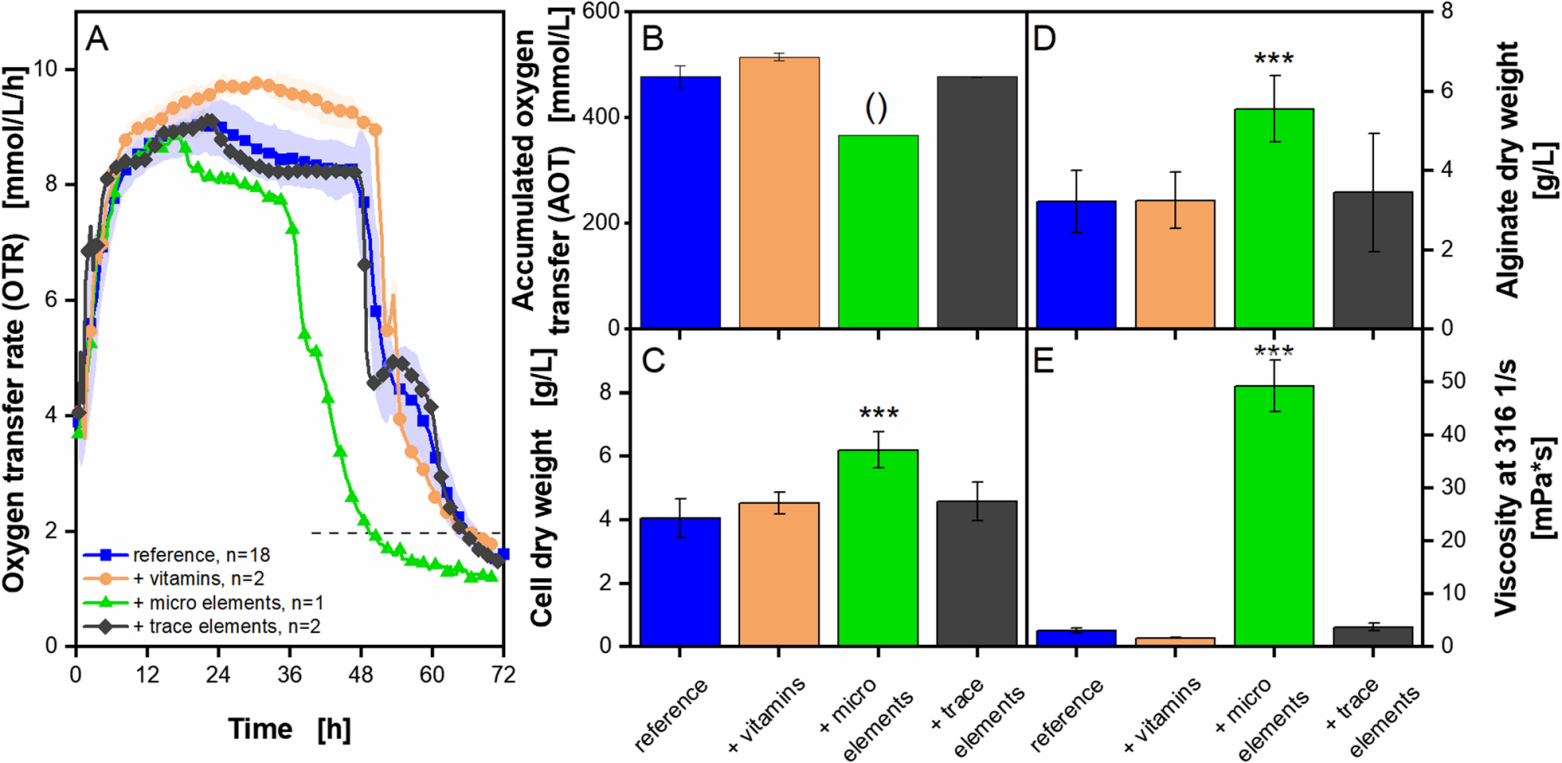
**Supplementation of vitamins, micro elements and trace elements to microaerobic** ***A. vinelandii* ATCC9046 cultivations**. Cultivations were conducted in modified Burk’s medium with 3 g/L yeast extract Roth Batch 1. Standard deviations are given for n ≥ 3 and are in some cases so small, that there are not well recognizable. Significant differences to the reference (3 g/L yeast extract) are marked with asterisks (*** for p < 0.001). If marked with ‘()’, no significance test could be conducted due to n = 1. **A**, **B**: For clarity, only every fifth measuring point of the OTR curves is shown. If n = 2, the mean value is shown and the shadow depicts the difference between the two replicates. The horizontal dashed line depicts the OTR value of 2 mmol/L/h for an easier comparison of the nominal cultivation time. **C**–**E**: All values are given as mean values, calculated from the following number of replicates: cell dry weight, n ≥ 8; alginate dry weight, n ≥ 6; broth viscosity, n ≥ 6. The flow curves, the mean molecular weight and the acetylation degree are provided as Additional file 1: Fig. S5. Cultivation conditions: 250 mL shake flasks, initial pH = 7.2, filling volume V_L_ = 50 mL, temperature T = 29 °C, shaking frequency n = 165 rpm, shaking diameter d_0_ = 50 mm

The exact composition of yeast extracts is most often not known, as manufacturers rarely provide detailed information on the exact composition of their products. Accordingly, it is not possible to easily attribute effects to specific chemical components. However, this information might be crucial, in order to supplement and improve poorer performing yeast extracts and to maintain production. Instead of performing an elaborate and expensive analysis of the chemical composition of a yeast extract, Diederichs et al. (2014) proposed to use screening methods, to identify suitable yeast extracts for a specific production system [26, 35, 90, 91]. This approach was also applied in this work. Certain supplements were added to the medium to elucidate, which components could possibly account for the differences between the yeast extracts. As the applied modified Burk’s medium is rather simple, with no added vitamins and only few trace elements (except those contained in yeast extract), the supplementation would possibly have the additional effect of improving growth and production. To investigate this strategy for *A. vinelandii* cultivations, the addition of a vitamin solution, a micro element solution and a trace element solution, originally from SYN6 medium [92], were investigated on their influence on *A. vinelandiis* growth and alginate production. These solutions were chosen as the first supplement, because SYN6 medium is rather rich, compared to other commonly used media, and, thus, provide a good basis for future experiments. The results of this supplementation experiment are presented in Fig. 5. As a reference, the poor performing yeast extract Roth Batch 1 was used, to detect, if supplementation would enhance its performance.

For the supplementation of trace elements, no change in OTR pattern, compared to the reference cultivation, could be observed (Fig. 5A). The addition of micro elements leads to a decrease in cultivation time by 13 h, compared to the reference cultivation. This corresponds with the calculated AOT, as it is only 0.7 times the reference value (Fig. 5B). The addition of the vitamin solution leads to a OTR plateau, that is at the highest level for a longer time compared to the other cultivations (Fig. 5A). For the addition of vitamins and micro elements, the shoulder in the OTR at the end of cultivation is less broad, compared to the reference cultivation.

The micro element solution is also the only supplement leading to an increase in cell dry weight, alginate dry weight and particularly in the culture broth viscosity (Fig. 5C–E). A final broth viscosity about sixteen times higher was obtained as a result of the addition of micro elements, and might result from the highest alginate concentration. The flow curves, plotted in Additional file 1: Fig. S5C, underline the results. Though, an additional influence on the viscosity by ions, provided by the micro element solution and interacting with the alginate, cannot be excluded.

In terms of the AOT, the results are rather counterintuitive. One would not expect the accumulated oxygen transfer to decrease, when an additional nutrient source is supplemented. Concomitantly, the biomass, alginate and broth viscosity increase. For the case of the mean molecular weight (Additional file 1: Fig. S5A), no significant differences compared to the reference could be observed, aside from the fact that higher mean molecular weights are reached by supplementing micro elements instead of vitamins. The acetylation degree of all supplemented cultivations shows no significant difference to the reference (Additional file 1: Fig. S5B).

The results above point towards a beneficial influence of supplementing micro elements to the modified Burk’s medium, as it enhances growth, production of alginate and, in particular, final broth viscosity. Furthermore, a different portfolio of micro elements may contribute to the differences observed between cultivations, when different yeast extracts were applied.

### Supplementation of copper, zinc and manganese sulphate to *A. vinelandii* cultivations under microaerobic conditions

**Fig. 6:**
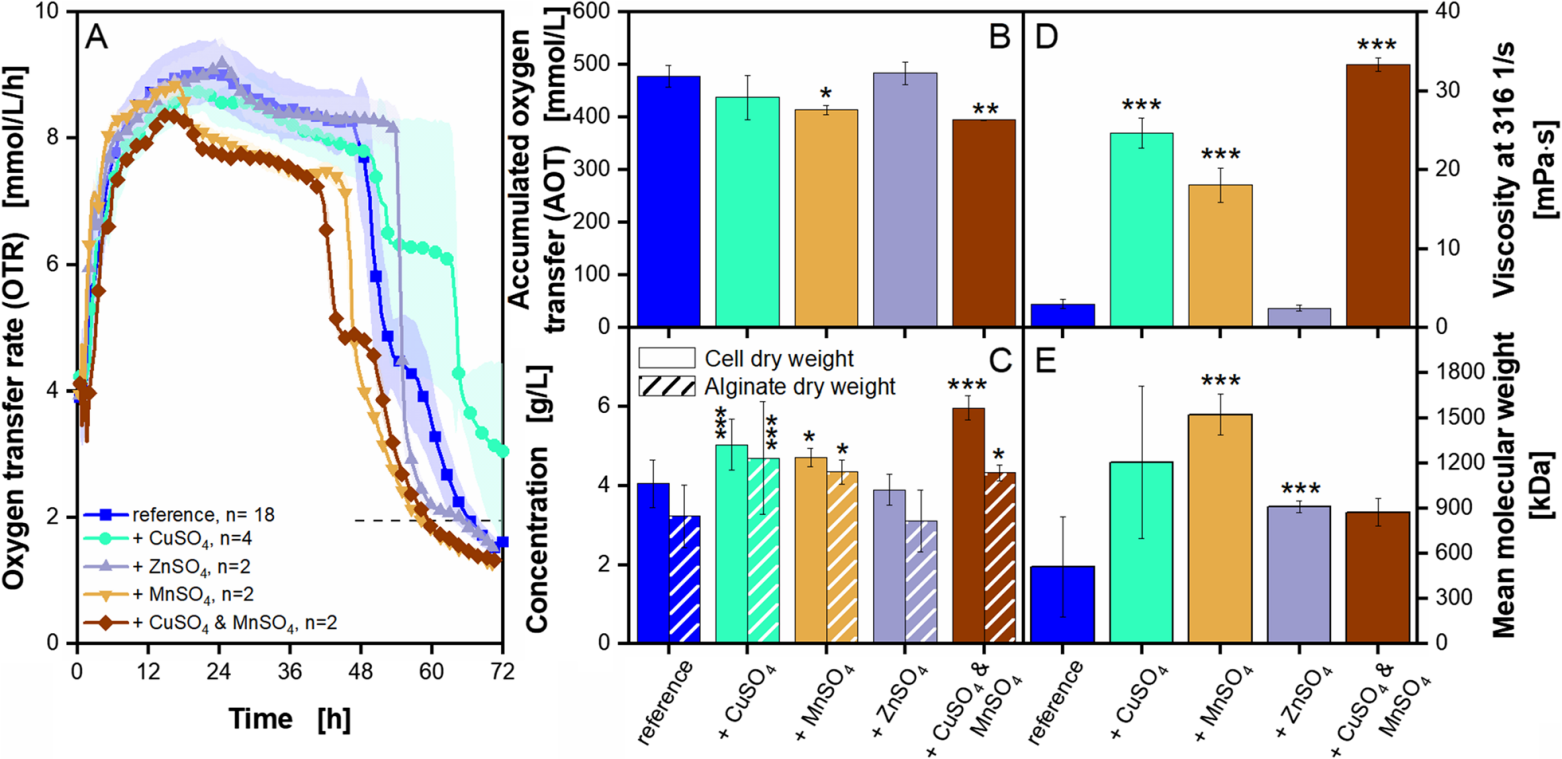
**Supplementation of copper, zinc and manganese sulfate to** ***A. vinelandii* ATCC9046 cultivations under microaerobic conditions**. Cultivations were conducted in modified Burk’s medium with 3 g/L yeast extract Roth Batch 1. Standard deviations are given for n ≥ 3. Significant differences to the reference are marked with asterisks, corresponding to the significance level (* for 0.01 < p < 0.05, ** for 0.001 < p < 0.01 and *** for p < 0.001). **A**, **B**: For clarity, only every fifth measuring point of the OTR curves is shown. The number of replicates is given with n; if n = 2, the mean value is shown and the shadow depicts the difference of the two replicates. In **A**, the horizontal dashed line depicts the OTR value of 2 mmol/L/h for an easier comparison of the nominal cultivation time. **C**–**E**: All values are given as mean values and calculated from the following number of replicates: cell dry weight, n ≥ 8; alginate dry weight, n ≥ 6; broth viscosity, n ≥ 4; mean molecular weight, n ≥ 6. The flow curves and the acetylation degree data are provided as Additional file 1: Fig. S6. Cultivation conditions: 250 mL shake flasks, initial pH = 7.2, filling volume V_L_ = 50 mL, temperature T = 29 °C, shaking frequency n = 165 rpm, shaking diameter d_0_ = 50 mm

In the previous section, the beneficial effect of the addition of micro elements on *A. vinelandii* cultivations were shown. The used micro element solution consists mainly of four components: copper, zinc, manganese and iron. To investigate, which of the micro elements led to the beneficial effect, they were supplemented separately to cultivations of *A. vinelandii*. However, iron is already present in the modified Burk’s medium in such high concentrations that it tends to precipitate and is, therefore, not supplemented separately. The results are presented in Fig. 6.

The most apparent effect of supplementation of single micro elements on the OTR can be observed with the supplementation of manganese sulphate (Fig. 6A). The addition leads to a shorter cultivation time by 8 h, compared to the reference. Furthermore, the cultivation with supplemented zinc sulphate is the only one without a shoulder formation. The reason for this phenomenon is up to now unclear. As the nitrogen source was not altered for the presented experiment, the shoulder formation possibly is not only dependent on the nitrogen availability, as stated by García et al. (2020) [62]. The cultivation with added manganese sulphate is, thus, the only one with a lower accumulated oxygen transfer (AOT) than the reference cultivation (Fig. 6B). The respiratory quotient (RQ) is available for this experiment, but no differences between the cultivations can be observed (Additional file 1: Fig. S6A).

The only cultivations reaching higher cell dry weight and alginate concentrations than the reference are the ones with added copper sulphate and manganese sulphate (Fig. 6C). For both cultivations, the viscosity is up to six to eight times higher than for the reference cultivation (Fig. 6D). This might originate in the elevated alginate concentrations. Additionally, the cultivation with added manganese sulphate shows an increased mean molecular weight of around 1500 kDa, which is 3 times higher than the reference cultivation (Fig. 6E). Interestingly, the addition of zinc sulphate leads to a small but significant increase in mean molecular weight, while all other investigated parameters seem to be not influenced by this addition. No significant differences for the acetylation degree can be observed (Additional file 1: Fig. S6B).

As the separate addition of copper and manganese sulphate led to the most promising results, both micro elements were simultaneously supplemented to one cultivation. The OTR in Fig. 6A shows a decrease of cultivation time by 7 h, compared to the reference cultivation. The AOT for this cultivation is even lower than the one with added manganese (Fig. 6B). The simultaneous addition of cooper and manganese sulphate leads to the highest biomass concentration, which is 1.5 times higher than the final concentration of the reference cultivation. The alginate concentration in comparable to the cultivation with added manganese sulphate (Fig. 6C). The broth viscosity is the highest with 33 mPa·s (Fig. 6D). The corresponding flow curves can be found in Additional file 1: Fig. S6C. Interestingly, the mean molecular weight was around 900 kDa lower than the cultivation with the addition of manganese (Fig. 6E). It is also noticeable that the separate supplementation of the micro elements does not lead to comparable results as the joint supplementation. For example, the broth viscosity with the addition of manganese is highest at 33 mPa·s, compared to the other individual supplemented elements (Fig. 6D). However, this is still lower than the 55 mPa·s, achieved with joint supplementation (Fig. 5E). The reason for this could be that the combination of elements is relevant for growth and production and influences the obtained concentrations and molecular properties.

The presented results do not allow for an in depth understanding of how the addition of certain micro elements influences the cells at a molecular level. The focus of this work was to demonstrate the general analysis method based on respiratory activity, growth and the molecular alginate properties. Nonetheless, the results are interesting and raise some questions regarding the complexity of *A. vinelandii* and its alginate production. Huyer and Page (1988) reported, that up to 200 µM zinc sulphate did not influence the growth of *A. vinelandii* [93]. In the present study, even a higher concentration of 12 mM showed no negative influence on growth or alginate production. It is known that Zn^+^ inhibits epimerases of *A. vinelandi*i, coded by the genes *algG* and *algE4* [94, 95]. However, as the content of monomers and their distribution in the molecule alginate were not investigated in this work, such an influence cannot be verified or excluded for the present experiments. This point would definitely be interesting to investigate in future works.

Ligand interaction of a number of enzymes, involved specifically in *A. vinelandii’s* alginate production, has not yet been fully characterized. Research on similar enzymes, sharing the same function, may serve as a basis for assumptions that need to be tested in the future. Inhibitory effects of zinc chloride, manganese chloride and copper sulphate have been reported for the GDP-mannose dehydrogenase, purified from two different algae species [96, 97]. This is partly in contrast to the results presented in this work. Bacteria exhibit resistance mechanisms towards metal ions, such as the ability to produce specific binding proteins. These have the purpose to buffer intrinsic metal ion levels and, hence, can save the cell and its enzymes from major damage or inhibition [98, 99]. Probably, the supplemented micro elements, coupled with the addition of EDTA, are in a concentration range that can be buffered and handled by *A. vinelandii*. In addition, the supplementation can even lead to beneficial effects on growth and alginate production.

### Supplementation of grouped amino acids to *A. vinelandii* cultivations

**Fig. 7:**
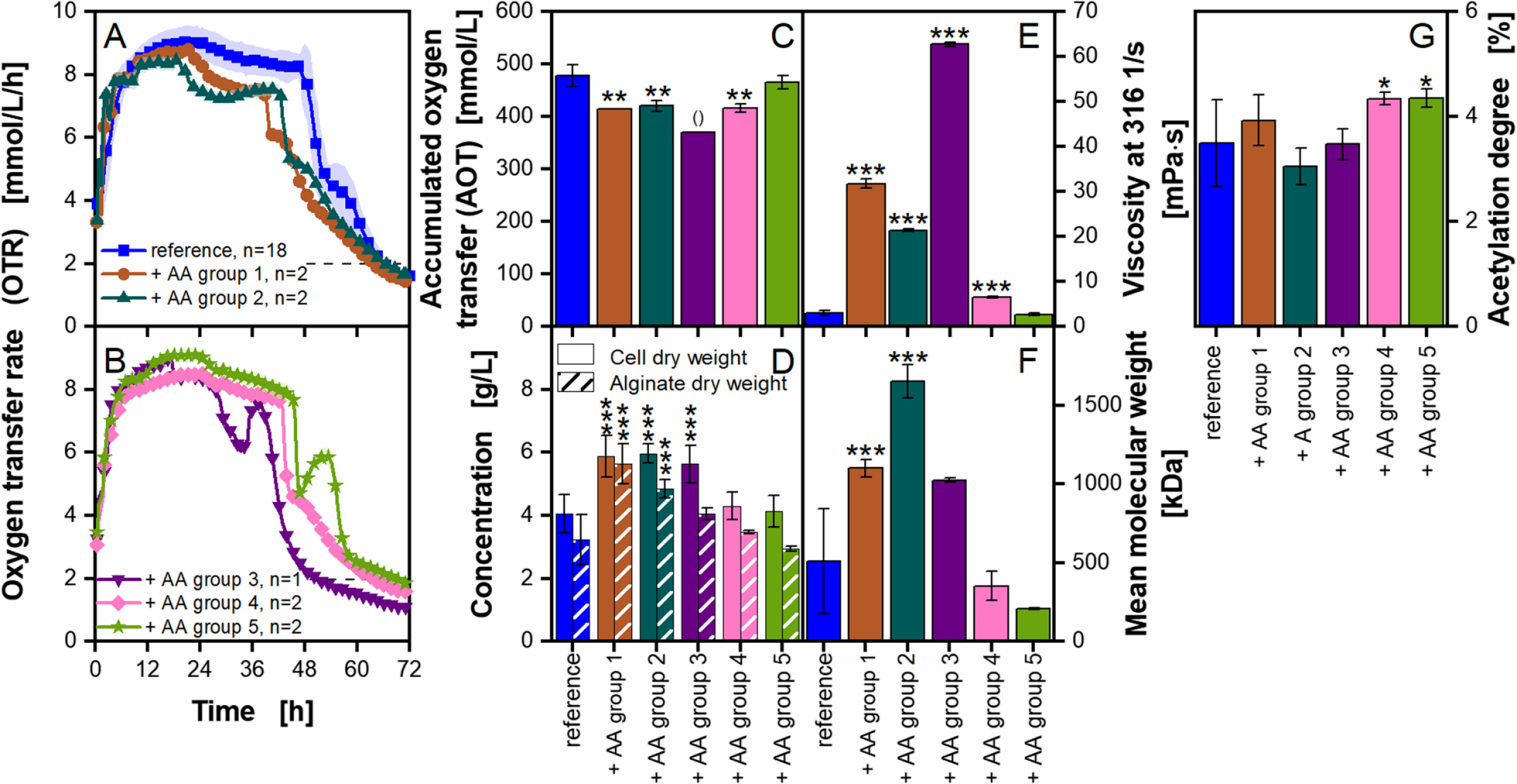
**Supplementation of grouped amino acids (AA) to** ***A. vinelandii* ATCC9046 cultivations under microaerobic conditions**. Cultivations were conducted in modified Burk’s medium with 3 g/L yeast extract Roth Batch 1. The composition of each amino acid group can be found in Table 2. Standard deviations are given for n ≥ 3. Significant differences to the reference (3 g/L yeast extract) are marked with asterisks, corresponding to the significance level (* for 0.01 < p < 0.05, ** for 0.001 < p < 0.01 and *** for p < 0.001). If marked with (), no significance tests could be conducted due to n = 1. **A**–**C**: For clarity, only every fifth measuring point of the OTR is shown. The number of replicates is given with n; if n = 2, the mean value is shown and the shadow depicts the difference of the two replicates. The horizontal dashed line depicts the OTR value of 2 mmol/L/h for an easier comparison of the nominal cultivation time. **D**–**G**: All values are given as mean values and were calculated from the following number of replicates: cell dry weight, n ≥ 4; alginate dry weight, n ≥ 3; broth viscosity, n ≥ 3; mean molecular weight and acetylation degree, n ≥ 3. The flow curves are provided as Additional file 1: Fig. S7C. Cultivation conditions: 250 mL shake flasks, initial pH = 7.2, initial pH for AA group 1 = 6.78, initial pH for AA group 2 & 4 = 6.95, filling volume V_L_ = 50 mL, temperature T = 29 °C, shaking frequency n = 165 rpm, shaking diameter d_0_ = 50 mm

A group of substances essential to bacteria are amino acids [100]. Several bacteria are capable of synthesizing all needed amino acids. Nevertheless, it has been reported that others are heterotrophic or face metabolic burdens, while producing such amino acids or proteins containing them [35, 55, 101–105]. Hence, the abundance or the accumulation of certain amino acids in different yeast extracts could lead to variations in microbial cultivation performance. To investigate this hypothesis, 18 amino acids in functional groups (Table 2), as presented by Müller et al. (2018) [55], were supplemented to *A. vinelandii* cultivations. Respiratory activity, growth and alginate production were measured. The results are presented in Fig. 7.

Figure 7A, B show the results of the OTR measurement. All cultivations with supplemented amino acids showed an earlier decrease from the OTR plateau towards the shoulder by 2 to 19 h, compared to the reference cultivation. The addition of amino acid groups 1, 2 and 4 leads to a weak shoulder formation. The addition of amino acid groups 3 and 5 leads to a more pronounced shoulder. Remarkably, the addition of amino acid group 3 shortens the cultivation time by 16 h. The AOT underlines those results (Fig. 7C). The addition of amino acid groups 1 to 4 leads to a lower AOT, and especially the AOT of cultivations with amino acid group 3 is only 0.7 times the AOT value of the reference cultivation. The RQ is similar for all cultivations, except for the cultivation with addition of group 3 (Additional file 1: Fig. S7A). A shift in the OTR and CTR caused the different RQ evolution. It cannot be excluded that this phenomenon might originate from a measurement error (Additional file 1: Fig. S7B). The cell dry weight (Fig. 7D) is increased up to 1.4 to 1.5 times for cultivations with the addition of group 1, 2 and 3. Only for the addition of group 1 and 2, an additional increase in alginate dry weight of up to 1.75 times is observable. Those three groups are also the groups showing a major increase in the broth viscosity at the end of cultivation (Fig. 7E). The addition of group 3 leads to an increased viscosity of 60 mPa·s, which is 20 times higher than the reference cultivation. Correspondingly, the flow curves show the same trends (Additional file 1: Fig. S7C). A slight, but significant increase in viscosity can be observed for the addition of amino acid group 4. The addition of amino acids group 1 and 2 leads to an increase in the mean molecular weight (Fig. 7F). Cultivations with group 2 reach around 1600 kDa. The addition of amino acids group 4 and 5 lead to elevated acetylation degrees compared to the reference. This might contribute to the fact, that the addition of group 4 reaches a higher final broth viscosity than the reference (Fig. 7E). The fact that the addition of group 4 leads to a slightly higher mean molecular weight than the addition of amino acid group 5 might explain, why a difference in the viscosity can be observed though similar acetylation degrees are reached. Interestingly, the cultivation with the highest broth viscosity (addition of amino acid group 3) is not the one cultivation, where the alginate produced had the highest mean molecular weight. Again, it is most likely that other molecular properties might be affected by the supplementation, such as the ratio of the two monomers guluronic acid and mannuronic acid or their distribution in the polymer. These parameters may enhance the viscosifying power of the alginate.

The most severe differences discussed above were caused by the addition of amino acids groups 1, 2 and 3. Interestingly, the addition of any amino acids did not change the OTR curves to an extent that would suggest the existence of a nutrient limitation or metabolic burden in the cultures without supplementation. Likewise, the change of OTR does not suggest that the amino acid amounts supplied via the yeast extracts were not sufficient to allow for sufficient growth. *A. vinelandii* is most likely not dependent on the supply of amino acids through yeast extracts, as the organism is most likely able to produce almost all of them on its own [106, 107]. Final sucrose concentrations were lower than 2% of the initial concentration for all cultivations (data not shown), again showing that the amount of amino acids provided via the yeast extract was not limiting the uptake of the main carbon source. The supplemented amino acids are most probably just adding additional amounts, to either enhance certain pathways for more growth or changing the flux within the cell towards a higher alginate production. The provision of certain amino acids could also enhance the production of enzymes located in the alginate production pathways and, hence, influencing e.g., the length of the polymer or its monomer content.

### Supplementation of single amino acids to *A. vinelandii* cultivations

**Fig. 8:**
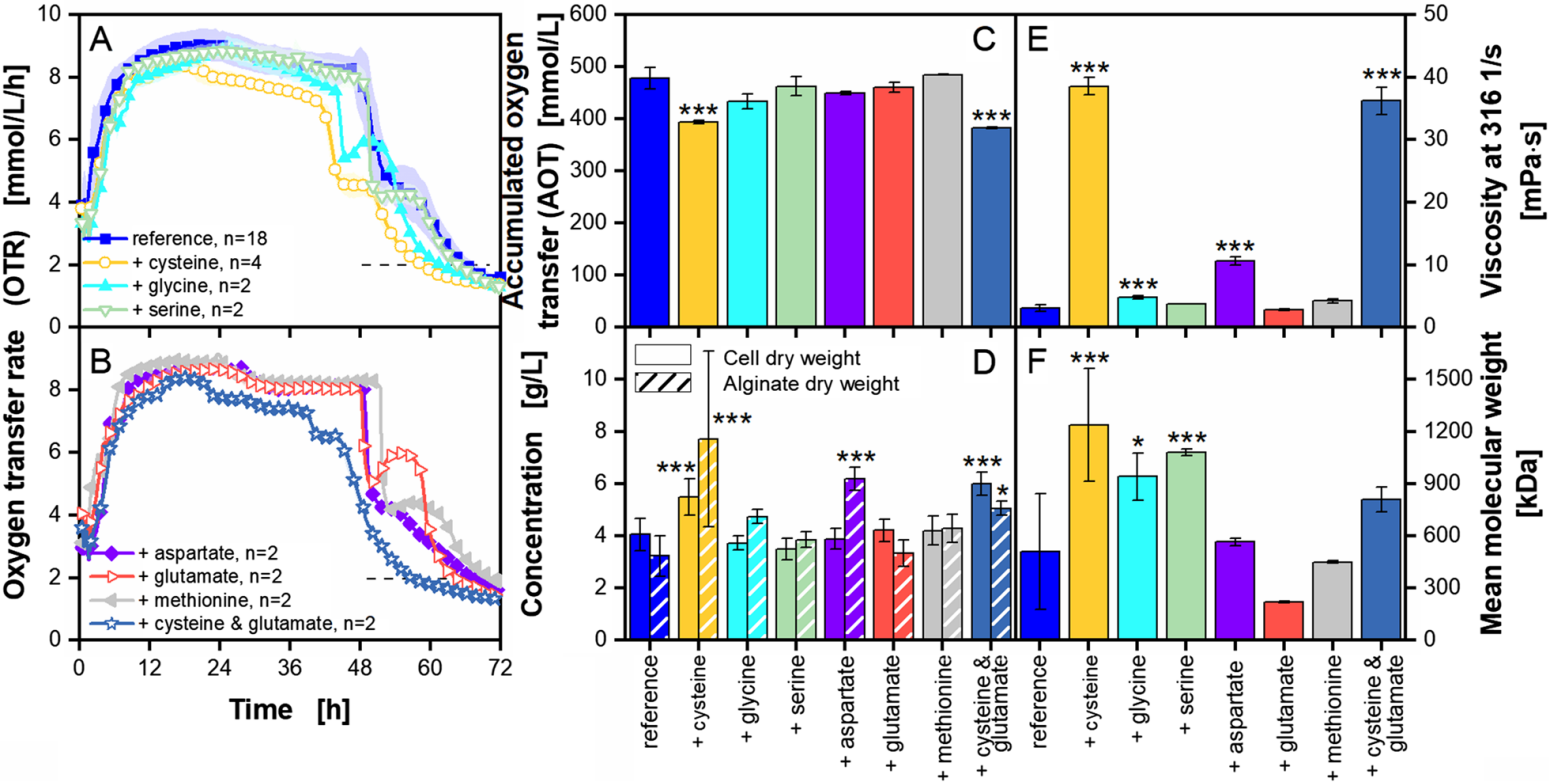
**Supplementation of single amino acids to** ***A. vinelandii* ATCC9046 cultivations under microaerobic conditions**. Cultivations were conducted in modified Burk’s medium with 3 g/L yeast extract Roth Batch 1. Standard deviations are given for n ≥ 3. Significant differences to the reference are marked with asterisks, corresponding to the significance level (* for 0.01 < p < 0.05, ** for 0.001 < p < 0.01 and *** for p < 0.001). **A**–**C**: For clarity, only every fifth measuring point of the OTR is shown. The number of replicates is given with n; if n = 2, the mean value is shown and the shadow depicts the difference of the two replicates. The horizontal dashed line depicts the OTR value of 2 mmol/L/h for an easier comparison of the nominal cultivation time. **D**–**F**: Values are given as mean values, calculated from the following number of replicates: cell dry weight from, n ≥ 8; alginate dry weight, n ≥ 6; broth viscosity, n ≥ 4; mean molecular weight, n ≥ 6. The flow curves and the acetylation degree data are provided as Additional file 1: Fig. S8. Cultivation conditions: 250 mL shake flasks, initial pH = 7.2, initial pH for supplementation of aspartate = 6.95, filling volume V_L_ = 50 mL, temperature T = 29 °C, shaking frequency n = 165 rpm, shaking diameter d_0_ = 50 mm

The addition of certain amino acids in groups led to improvements of the cultivation performance of *A. vinelandii*, including more growth and a higher alginate production with a higher molecular weight (Fig. 7). For a deeper investigation, selected amino acids from groups with the most pronounced influences were chosen for single supplementation. The decision was further based on the basic amino acid pathways, to cover different entries into the metabolism, while keeping the experimental effort in a manageable way. The results of the supplementation of single amino acids are presented in Fig. 8.

Figure 8A shows the OTR for all amino acids of group 3. The addition of glycine leads to a higher shoulder formation between 48 and 60 h, compared to the reference. The addition of cysteine leads to the lowest cultivation time, which is 8 h shorter than the reference cultivation. Adding serine, aspartate or methionine to the cultivation seems to have no effect on the OTR (Fig. 8B). Comparable to the addition of glycine, the addition of glutamate leads to a shoulder between 50 and 63 h at a higher OTR level than the reference cultivation. Additionally, the combined supplementation of cysteine and glutamate leads to a 10 h shorter cultivation time compared to the addition of cysteine alone. Furthermore, the shoulder formation is less pronounced than for cultivations with the addition of glutamate alone. No differences in the RQ (Additional file 1: Fig. S8A) between the cultivations can be noticed. The shorter cultivation times are reflected in the AOT (Fig. 8C). The addition of cysteine, alone or combined with glutamate, leads to an AOT, that is only 0.8 times the AOT value of the reference cultivation. These supplementations increase cell dry weight by 1.4 to 1.5 times and alginate dry weight by 1.6 to 2.4 times. The cultivation with aspartate shows a 1.9-fold increase in alginate dry weight. This result is reflected in the broth viscosity in Fig. 8E. The broth viscosity for cultivations with aspartate reaches values around 10 mPa s, which is 3.5 times higher than the reference. Even higher viscosities are reached with the addition of cysteine at around 40 mPa s. The additional supplementation of glutamate does not lead to higher viscosities. These findings are supported by the flow curves in Additional file 1: Fig. S8B. Interestingly, cultivations with added glutamate show a more pronounced shear thinning behaviour than other low-viscosity cultivations. Furthermore, a small increase in viscosity compared to the reference, can be observed for cultivations with added glycine. The mean molecular weight of the produced alginate is half as big for cultivations with the combined supplementation of cysteine and glutamate, as for the cultivations with the addition of cysteine alone (Fig. 8F). The addition of serine and glycine separately also leads to higher mean molecular weight of 943 to 1083 kDa. No significant differences of the acetylation degree to the reference could be observed (Additional file 1: Fig. S8C). Nonetheless, the acetylation degree is lower for cultivations with the addition of aspartate, glutamate and methionine compared to the addition of glycine, serine or the combination of cysteine and glutamate.

In comparison to the grouped supplementation, it can be concluded that no single amino acid supplementation led to exactly same results as if supplemented within its amino acid group. Certain parameters, such as those of the mean molecular weights of the alginates resulting from cultivations with cysteine or amino acid group 3, are comparable. Nevertheless, these cultivations show then different broth viscosities. The monomer content and the sequence in the macromolecule, that were not investigated within this work, might explain the observed differences. Thus, the influences observed on growth and alginate production are affected by the type of amino acid supplemented. Combined supplementation leads to different results. Hence, differences in the performance of yeast extracts can also be explained by a varying amino acid portfolio. As the influences vary depending on the combination of supplementation, these can be used to manipulate alginate production, to meet certain product requirements.

### Supplementation of combinations of micro elements and amino acids to *A. vinelandii* cultivations

**Fig. 9:**
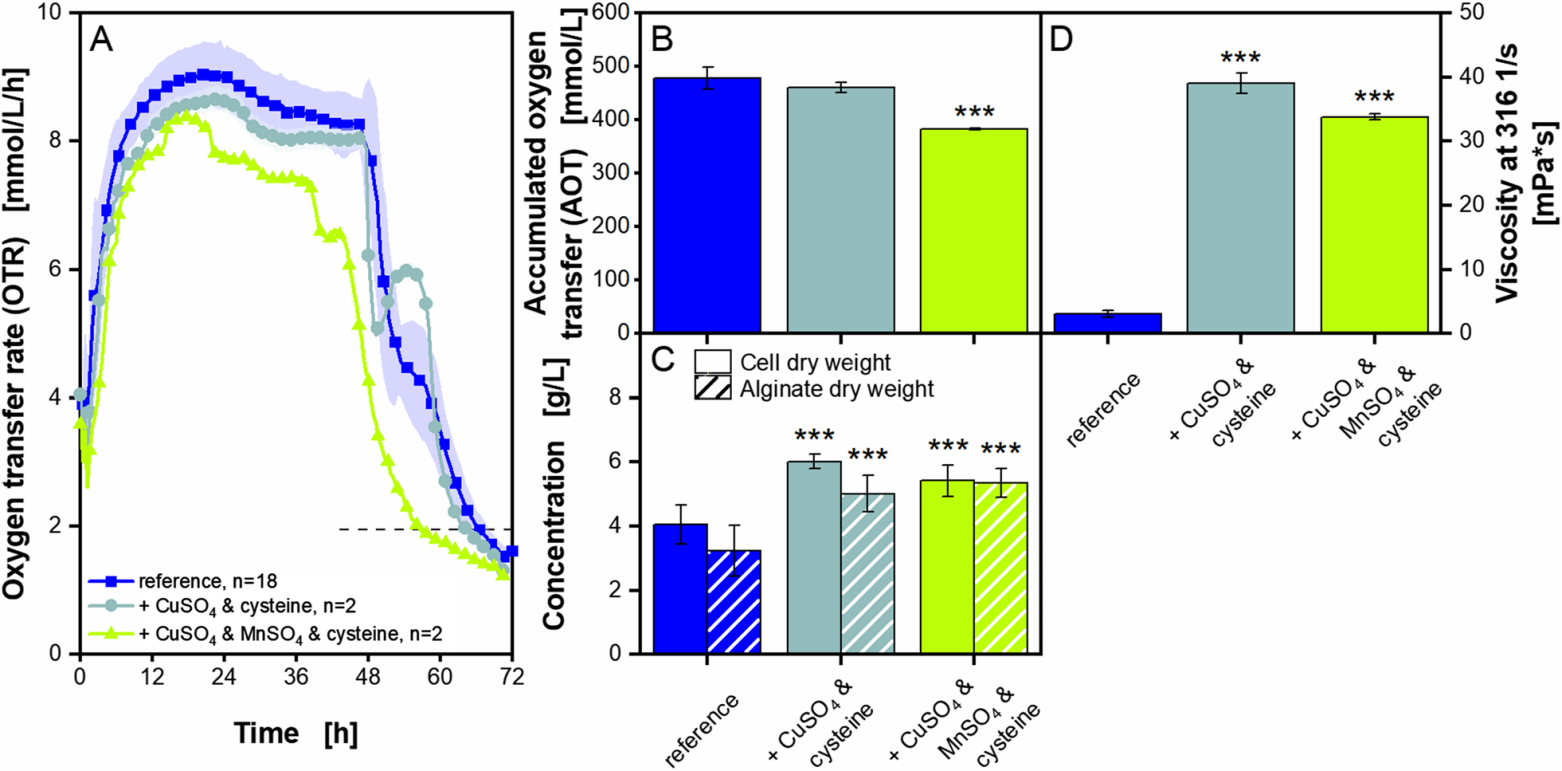
**Supplementation of combinations of micro elements and amino acids to microaerobic** ***A. vinelandii* ATCC9046 cultivations**. Cultivations were conducted in modified Burk’s medium with 3 g/L yeast extract Roth Batch 1. Standard deviations are given for n ≥ 3. Significant differences to the reference (3 g/L yeast extract) are marked with asterisks, corresponding to the significance level (*** for p < 0.001). **A**, **B**: For clarity, only every fifth measuring point of the OTR is shown. The number of replicates is given with n; if n = 2, the mean value is shown and the shadow depicts the difference of the two replicates. The horizontal dashed line depicts the OTR value of 2 mmol/L/h for an easier comparison of the nominal cultivation time. **C**, **D**: Values are given as mean values, calculated from the following number of replicates: cell dry weight, n ≥ 8, alginate dry weight, n ≥ 6; broth viscosity, n ≥ 4. The flow curves, the mean molecular weight and the acetylation degree are provided as Additional file 1: Fig. S9. Cultivation conditions: 250 mL shake flasks, initial pH = 7.2, filling volume V_L_ = 50 mL, temperature T = 29 °C, shaking frequency n = 165 rpm, shaking diameter d_0_ = 50 mm

Certain micro elements and amino acids investigated within this study showed a positive effect on *A. vinelandiis* growth and alginate production. To investigate, if an even more increased positive effect can be provoked, chosen micro elements and amino acids were combined. Therefore, cultivations with the combined supplementation of copper sulphate and cysteine as well as copper sulphate, manganese sulphate and cysteine were conducted. The results are presented in Fig. 9.

The addition of copper sulphate and cysteine leads for 48 h to a comparable OTR evolution, as well as a similar cultivation time, as observed for the reference cultivation (Fig. 9A). But the shoulder after 48 h for the supplemented cultivation with copper sulphate and cysteine is more pronounced. The supplementation with copper and manganese sulphate as well as cysteine leads to a decrease in cultivation time by around 12 h, compared to the reference. For this cultivation with three supplements, the shoulder formation at around 40 h is less pronounced than the shoulder formations of the two other presented cultivations. This again indicates that the shoulder formation is most likely not only connected to the availability of nitrogen, as stated by García et al. (2020) [62]. All cultivations show a similar evolution of the RQ (Additional file 1: Fig. S9A). The AOT for the cultivation with supplemented copper sulphate and cysteine is comparable to the reference (Fig. 9B). The AOT for the cultivation with supplemented copper and manganese sulphate as well as cysteine is around 0.7 times the value of the reference cultivation. Furthermore, both supplementations increase the cell dry weight by up to 1.5 times and the alginate dry weight by up to 1.7 times (Fig. 9C). The increased alginate concentration is related with the broth viscosity (Fig. 9D). The addition of copper sulphate and cysteine leads to a broth viscosity of around 40 mPa·s, which is 13 times higher than the final broth viscosity of the reference cultivation. Interestingly, the additional supplementation of manganese sulphate does not increase the broth viscosity further. The results correspond to the flow curve measurement, depicted in Additional file 1: Fig. S9B. For the mean molecular weight as well as the acetylation degree, no significant differences to the reference cultivation could be identified (Additional file 1: Fig. S9C, D).

If compared to previous experiments with single substance supplementation, it can be stated that supplementing more than one single substance does not always lead to better results. The mean molecular weight is around 400 kDa lower for the combined supplementations presented in Fig. 9, compared to supplementation of copper and manganese sulphate as well as cysteine separately (Fig. 6E for copper and manganese sulphate and Fig. 8F for cysteine). On the other hand, in case of copper sulphate (Fig. 6D), the broth viscosity is higher, if combined with cysteine (Fig. 9D). The results show that supplementation with its effects on growth and alginate production of *A. vinelandii* is rather complex. Nonetheless, the combined or separate supplementation of copper and manganese sulphate as well as cysteine shows an increase for some parameters, comparable to other yeast extracts. For example, cultivations with Merck yeast extract were considered well performing, as high broth viscosities of around 54 mPa·s, high molecular weights of approximately 1150 kDa and elevated concentration levels of cell and alginate dry weight of 7.7 and 4.7 g/L, respectively, were reached (Fig. 3E–H). The supplementation of cysteine, alone or in combination with copper sulphate, to cultivations conducted with Roth Batch 1 yeast extract, leads to viscosities around 40 mPa·s (Figs. 8E and 9D). The separate supplementation of cysteine and manganese sulphate leads to mean molecular masses of around 1200 kDa and even 1500 kDa (Figs. 8F and 6E). And the addition of cysteine alone (Fig. 8D) or in combination with either copper sulphate or copper and manganese sulphate (Fig. 9C) increases the concentration levels of cell dry weight and alginate, compared to the reference. The supplementation of the named substances to cultivations with the poorly-performing yeast extract Roth Batch 1 can thus be improved to reach values comparable to better-performing yeast extracts, such as from the manufacturer Merck. Hence, those substances, namely copper and manganese sulphate as well as cysteine, may play a major role in how yeast extracts influence cultivations. An overview of which yeast extracts and supplementations lead to particularly good results for each analysed parameter, can be found in Additional file 1: Table S1.

### Correlating viscosity and accumulated oxygen transfer

**Fig. 10:**
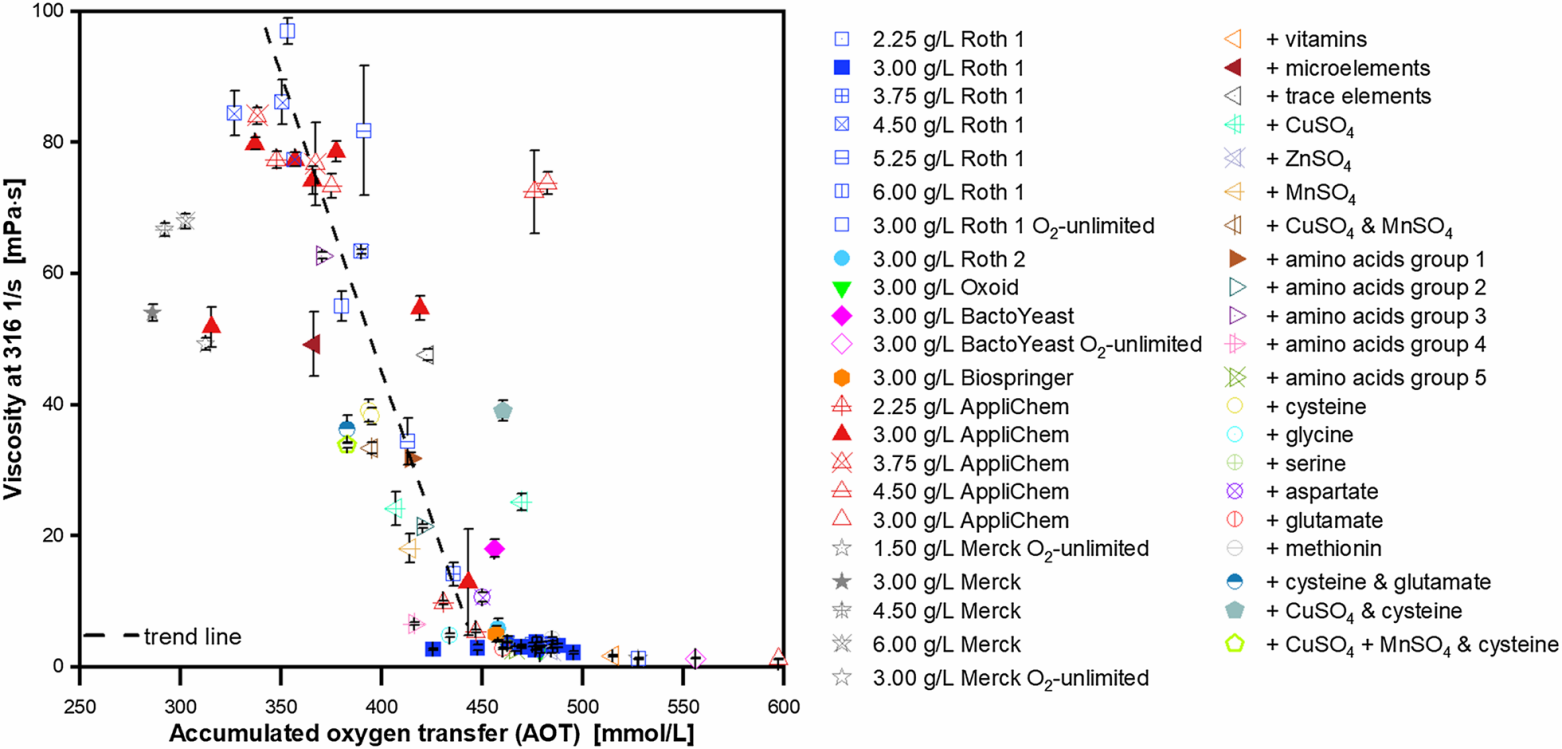
**Correlation of final broth viscosity and accumulated oxygen transfer of all presented** ***A. vinelandii* cultivations**. *A. vinelandii* ATCC9046 was cultivated in modified Burk’s medium with varying concentrations and suppliers of yeast extract as well as different supplements. If not stated otherwise in the legend of this diagram, 3 g/L yeast extract from Roth Batch 1 were used. The standard deviation was calculated for n ≥ 3. Cultivation conditions for microaerobic conditions: 250 mL shake flasks, filling volume V_L_ = 50 mL, temperature T = 29 °C, shaking frequency n = 165 rpm, shaking diameter d_0_ = 50 mm, initial pH = 7.2, initial pH for AA group 1 = 6.78, initial pH for AA group 2 & 4 and supplementation of aspartate = 6.95. Cultivation conditions for oxygen non-limiting conditions: 250 mL shake flasks, filling volume V_L_ = 10 mL, temperature T = 29 °C, shaking frequency n = 350 rpm, shaking diameter d_0_ = 50 mm, initial pH = 7.2

The results of experiments with different yeast extracts, including also the results of experiments with supplementation, showed that, depending on the condition, the cultivation time can vary. Since all cultivations reached similar OTR plateaus, due to the chosen shaking parameters (microaerobic conditions), the decrease in cultivation time led to a decrease in the AOT. In almost all experiments, the AOT decrease was accompanied by increases of biomass and alginate production, mean molecular weight and/or broth viscosity. To verify whether or not there is a correlation, the broth viscosity at a shear rate of 316 1/s is shown in Fig. 10 as a function of the AOT.

The results of all presented experiments were included. All microaerobic cultivations were conducted using the same shaking parameters. Although the data shows some scatter, a clear trend is recognizable. This trend is illustrated by a dashed line. The lower the AOT, the higher is the final viscosity of a cultivation. This is an interesting observation. Presumably, the addition of specific supplements cancels out an existing deficiency in the yeast extract. This deficiency may have limited the bacteria and the metabolic activity declines. The removal of the deficiency results in increased activity of the microorganisms. As expected, oxygen consumption should increase. Since this is obviously not the case, this hypothesis is not applicable for the presented results. Alternatively, it can be assumed that the removal of the deficiency results in a fundamental shift in the use of the carbon source. Hence, more biomass and/or alginate are produced from the provided carbon source and less carbon is burned for maintenance. Accordingly, the oxygen consumption declines and leads to a lower AOT. It is also possible that certain supplements additionally influence the activity of certain enzymes, so that the molecular properties of the alginate vary. Increased alginate concentrations or certain molecular properties, such as a higher molecular weight, then lead to an increase in the viscosity of the culture broth. Various enzymes are involved in the production of alginate and are responsible for its molecular properties. To date, not all enzymes have been identified and characterised in detail, especially for *A. vinelandii*. Part of the knowledge is based on the findings obtained with *Pseudomonas aeruginosa*, a bacterium that also produces alginate and whose biosynthesis is thought to be similar to the biosynthesis in *A. vinelandii* [68, 108, 109]. A lot of enzymes and regulatory proteins are involved, whose formation and function can also be influenced by several factors, such as cultivation parameters or media components [52, 110]. The biosynthesis and regulation of alginate are complex [111–113]. Accordingly, it is difficult to identify how and by what means they are influenced. Nevertheless, it is clear that different media components may well have an influence on the regulatory network and enzymatic activity in some way. Due to so many regulatory target points, the results can be very different, when it comes to the parameters mean molecular weight, acetylation or monomer content, which all have an impact on the molecular properties of the biopolymer alginate.

The exact effects of the individual supplements at the molecular level could not be determined within the scope of this work. However, the results suggest that the shift in the use of the carbon source, accompanied by changes in the activity of enzymes responsible for the molecular properties, is the most probable hypothesis to explain the obtained results. It also has to be kept in mind, that there might not be only one mechanism that applies for the effect of each individual supplementation. Presumably, the molecular mechanisms may vary from supplement to supplement. A correlation of viscosity and respiratory activity has been demonstrated. Therefore, it is unlikely that ions added by the supplementation to the medium and interacting with the polymer, are the only reason for elevated viscosities, as previously discussed.

## Conclusion

This study investigated the impact of yeast extracts of different manufacturers, lots from the same manufacturer and specific supplements on their influence on *A. vinelandii* cultivations, regarding respiratory activity, growth and alginate production. The results show that the choice of yeast extract has a major influence on the investigated parameters. Yeast extracts with poor performance can be improved through targeted supplementation and achieve similar quality attributes as those with good performance without supplements. A combination of micro elements including copper, manganese and zinc sulphate was investigated, as well as grouped amino acids. Promising components were then added separately. Especially the supplementation of cysteine, copper sulphate and manganese sulphate, alone or combined, shows beneficial effects on the above-mentioned quality attributes. On the one hand, the results show, which compounds contribute to the differences between the yeast extracts and how the experimental results can be improved with targeted supplements, in order to meet specific product requirements. At the same time, the results also demonstrate the potential of a media optimisation. In future studies, it should be considered to specifically analyse the yeast extracts for their content of cysteine, copper and manganese sulphate, to verify the results obtained in this work.

Based on the experimental results, a correlation between the final broth viscosity (at a shear rate of 316 1/s) and the AOT was unveiled. The lower the AOT, the higher is the viscosity. In the future, this correlation needs to be supported with further data. At this point, the correlation allows for a quick assessment of a cultivation, based on online-monitoring of the OTR. Thus, it can be used to estimate, whether a cultivation might lead to a higher viscosity and to elevated levels of either alginate concentration, its molecular properties, or even both, compared to another cultivation. Still, the underlying molecular effects of the observed results are to this point not understood and need to be investigated with more detail in the future.

## Supplementary Information

**Additional file 1: Table S1.** Conditions to be selected to achieve desired final values for selected cultivation features. **Figure S1.** Oxygen transfer rates of microaerobic *A. vinelandii* ATCC9046 cultivations with two different yeast extracts. **Figure S2.** Effect of different yeast extracts on *A. vinelandii* ATCC9046 cultivations at sufficient oxygen supply. **Figure S3.** Acetylation degree and final broth viscosity of *A. vinelandii *ATCC9046 cultivations with different yeast extracts. **Figure S4.** Final broth viscosity of microaerobic *A. vinelandii* ATCC9046 cultivations with varying yeast extract concentrations. **Figure S5.** Further analytics of *A. vinelandii* ATCC9046 cultivations, supplemented with vitamins, micro elements and trace elements. **Figure S6.** Further analytics of *A. vinelandii* ATCC9046 cultivations with supplementation of copper, zinc and manganese sulfate. **Figure S7.** Further analytics of microaerobic *A. vinelandii* ATCC9046 cultivations with supplementation of different grouped amino acids. **Figure S8.** Further analytics of microaerobic *A. vinelandii* ATCC9046 cultivations with supplementation of single amino acids. **Figure S9.** Further analytics of *A. vinelandii* ATCC9046 cultivations with supplementation of micro elements and amino acids.

## Data Availability

The datasets used and analysed during the current study are available from the corresponding author on reasonable request.

## Abbreviations

AC: Applichem
AOT: Accumulated oxygen transfer
BS: BioSpringer
BY: BactoYeast
CTR: Carbon dioxide transfer rate
EDTA: Ethylenediaminetetraacetic acid
GDP: Guanosine diphosphate
M: Merck
MMW: Mean molecular weight
MOPS: 3-(N-morpholino)propanesulfonic acid)
O: Oxoid
OTR: Oxygen transfer rate
OTR_max_: Maximum oxygen transfer capacity
R-1: Roth batch 1
R-2: Roth batch 2
RQ: Respiratory quotient

## References

[1] Draget KI, Taylor C (2011). Chemical, physical and biological properties of alginates and their biomedical implications. Food Hydrocolloid.

[2] Liu J, Yang SQ, Li XT, Yan QJ, Reaney MJT, Jiang ZQ (2019). Alginate oligosaccharides: production, biological activities, and potential applications. Compr Rev Food Sci F.

[3] Chowdhury S, Chowdhury IR, Kabir F, Mazumder MA, Zahir MH, Alhooshani K (2019). Alginate-based biotechnology: a review on the arsenic removal technologies and future possibilities. J Water Supply Res T.

[4] Wawrzynska E, Kubies D (2018). Alginate matrices for protein delivery—a short review. Physiol Res.

[5] Qin Y, Jiang J, Zhang J, Wang F (2018). Applications of alginate as a functional food ingredient BioPolymers for food design.

[6] Cikrikci S, Mert B, Oztop MH (2018). Development of pH sensitive alginate/gum tragacanth based hydrogels for oral insulin delivery. J Agr Food Chem.

[7] Sun JC, Tan HP (2013). Alginate-based biomaterials for regenerative medicine applications. Materials.

[8] Lee KY, Mooney DJ (2012). Alginate: properties and biomedical applications. Prog Polym Sci.

[9] Clementi F, Mancini M, Moresi M (1998). Rheology of alginate from *Azotobacter vinelandii* in aqueous dispersions. J Food Eng.

[10] Clementi F (1997). Alginate production by *Azotobacter vinelandii*. Crit Rev Biotechnol.

[11] Gacesa P (1998). Bacterial alginate biosynthesis—recent progress and future prospects. Microbiol-Sgm.

[12] Galindo E, Peña C, Núñez C, Segura D, Espin G (2007). Molecular and bioengineering strategies to improve alginate and polydydroxyalkanoate production by *Azotobacter vinelandii*. Microb Cell Fact.

[13] Castillo T, Heinzle E, Peifer S, Schneider K, Peña CF (2013). Oxygen supply strongly influences metabolic fluxes, the production of poly(3-hydroxybutyrate) and alginate, and the degree of acetylation of alginate in *Azotobacter vinelandii*. Process Biochem.

[14] Segura D, Guzmaan J, Espin G (2003). *Azotobacter vinelandii* mutants that overproduce poly-beta-hydroxybutyrate or alginate. Appl Microbiol Biot.

[15] Jimenez L, Castillo T, Flores C, Segura D, Galindo E, Peña C (2016). Analysis of respiratory activity and carbon usage of a mutant of *Azotobacter vinelandii* impaired in poly-beta-hydroxybutyrate synthesis. J Ind Microbiol Biot.

[16] Cunha CCF, Glassey J, Montague GA, Albert S, Mohan P (2001). Incorporation of raw material quality information on fermentation estimation models. IFAC Proc Vol.

[17] Dahod SK, Greasham R, Kennedy M, Baltz Richard H, Davies Julian E, Demain Arnold L, Bull Alan T, Junker Beth, Katz Leonard, Lynd Lee R, Masurekar Prakash, Reeves Christopher D, Zhao Huimin (2010). Raw materials selection and medium development for industrial fermentation processes. Manual of industrial microbiology and biotechnology.

[18] Rathore AS, Bhambure R, Ghare V (2010). Process analytical technology (PAT) for biopharmaceutical products. Anal Bioanal Chem.

[19] Services USDoHaH, Administration FaD. PAT - A framework for innovative pharmaceutical development, manufacturing, and quality assurance: FDA; 2004. https://www.fda.gov/regulatory-information/search-fda-guidance-documents/pat-framework-innovative-pharmaceutical-development-manufacturing-and-quality-assurance.

[20] Chopda V, Gyorgypal A, Yang O, Singh R, Ramachandran R, Zhang HR (2021). Recent advances in integrated process analytical techniques, modeling, and control strategies to enable continuous biomanufacturing of monoclonal antibodies. J Chem Technol Biot.

[21] Kirdar AO, Chen G, Weidner J, Rathore AS (2010). Application of near-infrared (NIR) spectroscopy for screening of raw materials used in the cell culture medium for the production of a recombinant therapeutic protein. Biotechnol Prog.

[22] Zhang JY, Reddy J, Buckland B, Greasham R (2003). Toward consistent and productive complex media for industrial fermentations: studies on yeast extract for a recombinant yeast fermentation process. Biotechnol Bioeng.

[23] Jacob FF, Hutzler M, Methner FJ (2019). Comparison of various industrially applicable disruption methods to produce yeast extract using spent yeast from top-fermenting beer production: influence on amino acid and protein content. Eur Food Res Technol.

[24] Verduyn C, Suksomcheep A, Suphantharika M (1999). Effect of high pressure homogenization and papain on the preparation of autolysed yeast extract. World J Microb Biot.

[25] Dimopoulos G, Tsantes M, Taoukis P (2020). Effect of high pressure homogenization on the production of yeast extract via autolysis and beta-glucan recovery. Innov Food Sci Emerg.

[26] Jacob FF, Striegel L, Rychlik M, Hutzler M, Methner FJ (2019). Yeast extract production using spent yeast from beer manufacture: influence of industrially applicable disruption methods on selected substance groups with biotechnological relevance. Eur Food Res Technol.

[27] Tanguler H, Erten H (2009). The effect of different temperatures on autolysis of baker's yeast for the production of yeast extract. Turk J Agric For.

[28] Inc. L. Bakers yeast production and characteristics. Lallemand Baking Update. 2018.

[29] Bekatorou A, Psarianos C, Koutinas AA (2006). Production of food grade yeasts. Food Technol Biotech.

[30] El-Helow ER, Elbahloul Y, El-Sharouny EE, Ali SR, Ali AAM (2015). Economic production of baker’s yeast using a new *Saccharomyces cerevisiae* isolate. Biotechnol Biotec Eq.

[31] Chapanya P, Ritthiruangdej P, Mueangmontri R, Pattamasuwan A, Vanichsriratana W (2019). Temperature compensation on sugar content prediction of molasses by near-infrared spectroscopy (NIR). Sugar Tech.

[32] Bortolussi G, O'Neill CJ (2006). Variation in molasses composition from eastern Australian sugar mills. Aust J Exp Agr.

[33] Vaisala. Application note—Yeast extract process. Food and beverage. 2020; 2.12.01. Available from: www.vaisala.com.

[34] Potvin J, Fonchy E, Conway J, Champagne CP (1997). An automatic turbidimetric method to screen yeast extracts as fermentation nutrient ingredients. J Microbiol Meth.

[35] Diederichs S, Korona A, Staaden A, Kroutil W, Honda K, Ohtake H (2014). Phenotyping the quality of complex medium components by simple online-monitored shake flask experiments. Microb Cell Fact.

[36] Fu XY, Wei DZ, Tong WY (2006). Effect of yeast extract on the expression of thioredoxin-human parathyroid hormone from recombinant *Escherichia coli*. J Chem Technol Biot.

[37] Sorensen JL, Sondergaard TE (2014). The effects of different yeast extracts on secondary metabolite production in *Fusarium*. Int J Food Microbiol.

[38] Iding K, Buntemeyer H, Gudermann F, Deutschmann SM, Kionka C, Lehmann J (2001). An automatic system for the assessment of complex medium additives under cultivation conditions. Biotechnol Bioeng.

[39] Zapata-Vélez AM, Trujillo-Roldán MA (2010). The lack of a nitrogen source and/or the C/N ratio affects the molecular weight of alginate and its productivity in submerged cultures of *Azotobacter vinelandii*. Ann Microbiol.

[40] Brivonese AC, Sutherland IW (1989). Polymer production by a mucoid strain of *Azotobacter vinelandii* in batch culture. Appl Microbiol Biot.

[41] Parente E, Crudele MA, Ricciardi A, Mancini M, Clementi F (2000). Effect of ammonium sulphate concentration and agitation speed on the kinetics of alginate production by *Azotobacter vinelandii* DSM576 in batch fermentation. J Ind Microbiol Biot.

[42] Revin VV, Kostina EG, Revina NV, Shutova VV (2018). Effect of nutrient sources on the alginate accumulation in the culture liquid of *Azotobacter vinelandii* D-05 and obtaining biocomposite materials. Braz Arch Biol Techn.

[43] Sabra W, Zeng AP, Sabry S, Omar S, Deckwer W-D (1999). Effect of phosphate and oxygen concentrations on alginate production and stoichiometry of metabolism of *Azotobacter vinelandii* under microaerobic conditions. Appl Microbiol Biotechnol.

[44] Peña C, Galindo E, Büchs J (2011). The viscosifying power, degree of acetylation and molecular mass of the alginate produced by *Azotobacter vinelandii* in shake flasks are determined by the oxygen transfer rate. Process Biochem.

[45] Sabra W, Zeng AP, Deckwer WD (2001). Bacterial alginate: physiology, product quality and process aspects. Appl Microbiol Biot.

[46] Horan NJ, Jarman TR, Dawes EA (1981). Effects of carbon source and inorganic phosphate concentration on the production of alginic acid by a mutant of *Azotobacter vinelandii* and on the enzymes involved in its biosynthesis. J Gen Microbiol.

[47] George SE, Costenbader CJ, Melton T (1985). Diauxic growth in *Azotobacter vinelandii*. J Bacteriol.

[48] Peña C, Hernández L, Galindo E (2006). Manipulation of the acetylation degree of *Azotobacter vinelandii* alginate by supplementing the culture medium with 3-(N-morpholino)-propane-sulfonic acid. Lett Appl Microbiol.

[49] Clementi F, Fantozzi P, Mancini F, Moresi M (1995). Optimal conditions for alginate production by *Azotobacter vinelandii*. Enzyme Microb Tech.

[50] Moral CK, Dogan O, Sanin FD (2015). Effect of oxygen tension and medium components on monomer distribution of alginate. Appl Biochem Biotech.

[51] Annison G, Couperwhite I (1986). Influence of calcium on alginate production and composition in continuous cultures of *Azotobacter vinelandii*. Appl Microbiol Biot.

[52] Mærk M, Jakobsen ØM, Sletta H, Klinkenberg G, Tøndervik A, Ellingsen TE (2020). Identification of regulatory genes and metabolic processes important for alginate biosynthesis in *Azotobacter vinelandii* by screening of a transposon insertion mutant library. Front Bioeng Biotech.

[53] Gomez-Pazarin K, Flores C, Castillo T, Büchs J, Galindo E, Peña C (2016). Molecular weight and viscosifying power of alginates produced in *Azotobacter vinelandii* cultures in shake flasks under low power input. J Chem Technol Biot.

[54] Keil T, Dittrich B, Lattermann C, Habicher T, Büchs J (2019). Polymer-based controlled-release fed-batch microtiter plate—diminishing the gap between early process development and production conditions. J Biol Eng.

[55] Müller J, Beckers M, Mussmann N, Bongaerts J, Büchs J (2018). Elucidation of auxotrophic deficiencies of *Bacillus pumilus* DSM 18097 to develop a defined minimal medium. Microb Cell Fact.

[56] Anderlei T, Büchs J (2001). Device for sterile online measurement of the oxygen transfer rate in shaking flasks. Biochem Eng J.

[57] Anderlei T, Zang W, Papaspyrou M, Büchs J (2004). Online respiration activity measurement (OTR, CTR, RQ) in shake flasks. Biochem Eng J.

[58] Regestein neé Meissner L, Arndt J, Palmen TG, Jestel T, Mitsunaga H, Fukusaki E (2017). Investigation of poly(gamma-glutamic acid) production via online determination of viscosity and oxygen transfer rate in shake flasks. J Biol Eng.

[59] Heyman B, Lamm R, Tulke H, Regestein L, Buchs J (2019). Shake flask methodolgy for assessing the influence of the maximum oxygen transfer capacity on 2,3-butanediol production. Microb Cell Fact.

[60] Antonov E, Wirth S, Gerlach T, Schlembach I, Rosenbaum MA, Regestein L (2016). Efficient evaluation of cellulose digestibility by *Trichoderma reesei* Rut-C30 cultures in online monitored shake flasks. Microb Cell Fact.

[61] Wewetzer SJ, Kunze M, Ladner T, Luchterhand B, Roth S, Rahmen N (2015). Parallel use of shake flask and microtiter plate online measuring devices (RAMOS and BioLector) reduces the number of experiments in laboratory-scale stirred tank bioreactors. J Biol Eng.

[62] García A, Castillo T, Ramos D, Ahumada-Manuel CL, Núñez C, Galindo E (2020). Molecular weight and viscosifying power of alginates produced by mutant strains of *Azotobacter vinelandii* under microaerobic conditions. Biotechnol Rep.

[63] Meier K, Klöckner W, Bonhage B, Antonov E, Regestein L, Büchs J (2016). Correlation for the maximum oxygen transfer capacity in shake flasks for a wide range of operating conditions and for different culture media. Biochem Eng J.

[64] Peña C, Campos N, Galindo E (1997). Changes in alginate molecular mass distributions, broth viscosity and morphology of *Azotobacter vinelandii* cultured in shake flasks. Appl Microbiol Biot.

[65] Giese H, Klöckner W, Peña C, Galindo E, Lotter S, Wetzel K (2014). Effective shear rates in shake flasks. Chem Eng Sci.

[66] Miller GL (1959). Use of dinitrosalicylic acid reagent for determination of reducing sugar. Anal Chem.

[67] Peña C, Trujillo-Roldan MA, Galindo E (2000). Influence of dissolved oxygen tension and agitation speed on alginate production and its molecular weight in cultures of *Azotobacter vinelandii*. Enzyme Microb Tech.

[68] Flores C, Díaz-Barrera A, Martinez F, Galindo E, Peña C (2015). Role of oxygen in the polymerization and de-polymerization of alginate produced by *Azotobacter vinelandii*. J Chem Technol Biot.

[69] Díaz-Barrera A, Maturana N, Pacheco-Leyva I, Martinez I, Altamirano C (2017). Different responses in the expression of alginases, alginate polymerase and acetylation genes during alginate production by *Azotobacter vinelandii* under oxygen-controlled conditions. J Ind Microbiol Biot.

[70] Peña C, Peter CP, Büchs J, Galindo E (2007). Evolution of the specific power consumption and oxygen transfer rate in alginate-producing cultures of *Azotobacter vinelandii* conducted in shake flasks. Biochem Eng J.

[71] Castillo T, López I, Flores C, Segura D, García A, Galindo E (2018). Oxygen uptake rate in alginate producer (algU+) and nonproducer (algU-) strains of *Azotobacter vinelandii* under nitrogen-fixation conditions. J Appl Microbiol.

[72] Llorens JMN, Tormo A, Martinez-Garcia E (2010). Stationary phase in gram-negative bacteria. Fems Microbiol Rev.

[73] Farias G, Fabregas E, Díaz-Barrera A, Ponce B, Castro C, Dormido-Canto S (2019). Automatic control for the production of alginate by *Azotobacter Vinelandii*. Ieee Access.

[74] Jensen HL (1954). The *Azotobacteriaceae*. Bacteriol Rev.

[75] Padilla-Cordova C, Mongili B, Contreras P, Fino D, Tommasi T, Díaz-Barrera A (2020). Productivity and scale-up of poly(3-hydroxybutyrate) production under different oxygen transfer conditions in cultures of *Azotobacter vinelandii*. J Chem Technol Biot.

[76] Oliveira CSS, Silva CE, Carvalho G, Reis MA (2017). Strategies for efficiently selecting PHA producing mixed microbial cultures using complex feedstocks: feast and famine regime and uncoupled carbon and nitrogen availabilities. New Biotechnol.

[77] Skjakbraek G, Zanetti F, Paoletti S (1989). Effect of acetylation on some solution and gelling properties of alginates. Carbohyd Res.

[78] Trujillo-Roldan MA, Moreno S, Espin G, Galindo E (2004). The roles of oxygen and alginate-lyase in determining the molecular weight of alginate produced by *Azotobacter vinelandii*. Appl Microbiol Biot.

[79] Meissner L, Kauffmann K, Wengeler T, Mitsunaga H, Fukusaki E, Büchs J (2015). Influence of nitrogen source and pH value on undesired poly(gamma-glutamic acid) formation of a protease producing *Bacillus licheniformis* strain. J Ind Microbiol Biot.

[80] Müller MJ, Stachurski S, Stoffels P, Schipper K, Feldbrügge M, Büchs J (2018). Online evaluation of the metabolic activity of *Ustilago maydis* on (poly)galacturonic acid. J Biol Eng.

[81] Herweg E, Schopping M, Rohr K, Siemen A, Frank O, Hofmann T (2018). Production of the potential sweetener 5-ketofructose from fructose in fed-batch cultivation with *Gluconobacter oxydans*. Bioresource Technol.

[82] Philip P, Kern D, Goldmanns J, Seiler F, Schulte A, Habicher T (2018). Parallel substrate supply and pH stabilization for optimal screening of *E. coli* with the membrane-based fed-batch shake flask. Microb Cell Fact.

[83] Kunze M, Huber R, Gutjahr C, Mullner S, Büchs J (2012). Predictive tool for recombinant protein production in *Escherichia coli* shake flask cultures using an on-line monitoring system. Biotechnol Progr.

[84] Palmen TG, Nieveler J, Frölich B, Treffenfeldt W, Pohl M, Büchs J (2010). Physiological relation between respiration activity and heterologous expression of selected benzoylformate decarboxylase variants in *Escherichia coli*. Microb Cell Fact.

[85] Fu S, Thacker A, Sperger DM, Boni RL, Buckner IS, Velankar S (2011). Relevance of rheological properties of sodium alginate in solution to calcium alginate gel properties. AAPS PharmSciTech.

[86] Fu S, Thacker A, Sperger DM, Boni RL, Velankar S, Munson EJ (2010). Rheological evaluation of inter-grade and inter-batch variability of sodium alginate. AAPS PharmSciTech.

[87] Rehm BHA, Valla S (1997). Bacterial alginates: biosynthesis and applications. Appl Microbiol Biot.

[88] Szekalska M, Pucilowska A, Szymanska E, Ciosek P, Winnicka K (2016). Alginate: current use and future perspectives in pharmaceutical and biomedical applications. Int J Polym Sci.

[89] Cho KS, Ryu HW, Park CH, Goodrich PR (2001). Utilization of swine wastewater as a feedstock for the production of polyhydroxyalkanoates by *Azotobacter vinelandii* UWD. J Biosci Bioeng.

[90] Kasprow RP, Lange AJ, Kirwan DJ (1998). Correlation of fermentation yield with yeast extract composition as characterized by near-infrared spectroscopy. Biotechnol Progr.

[91] Li BY, Sirimuthu NMS, Ray BH, Ryder AG (2012). Using surface-enhanced Raman scattering (SERS) and fluorescence spectroscopy for screening yeast extracts, a complex component of cell culture media. J Raman Spectrosc.

[92] Jeude M, Dittrich B, Niederschulte H, Anderlei T, Knocke C, Klee D (2006). Fed-batch mode in shake flasks by slow-release technique. Biotechnol Bioeng.

[93] Huyer M, Page WJ (1988). Zn^2+^ increases siderophore production in *Azotobacter vinelandii*. Appl Environ Microb.

[94] Rehm BH, Ertesvag H, Valla S (1996). New *Azotobacter vinelandii* mannuronan C-5-epimerase gene (algG) is part of an alg gene cluster physically organized in a manner similar to that in *Pseudomonas aeruginosa*. J Bacteriol.

[95] Hoidal HK, Ertesvag H, Skjak-Braek G, Stokke BT, Valla S (1999). The recombinant *Azotobacter vinelandii* mannuronan C-5-epimerase AlgE4 epimerizes alginate by a nonrandom attack mechanism. J Biol Chem.

[96] Tenhaken R, Voglas E, Cock JM, Neu V, Huber CG (2011). Characterization of GDP-mannose dehydrogenase from the brown alga *Ectocarpus siliculosus* providing the precursor for the alginate polymer. J Biol Chem.

[97] Zhang PY, Shao ZR, Jin WH, Duan DL (2016). Comparative characterization of two GDP-mannose dehydrogenase genes from *Saccharina japonica* (*Laminariales, Phaeophyceae*). Bmc Plant Biol.

[98] Choudhury R, Srivastava S (2001). Zinc resistance mechanisms in bacteria. Curr Sci India.

[99] Ladomersky E, Petris MJ (2015). Copper tolerance and virulence in bacteria. Metallomics.

[100] Tomita T (2017). Structure, function, and regulation of enzymes involved in amino acid metabolism of bacteria and archaea. Biosci Biotech Bioch.

[101] Zhou YX, Shen P, Lan QY, Deng C, Zhang Y, Li YC (2017). High-coverage proteomics reveals methionine auxotrophy in *Deinococcus radiodurans*. Proteomics.

[102] Heizer EM, Raymer ML, Krane DE, Raiford DW. Perceived cost of auxotrophic amino acids in two bacterial species. 2009 Ohio Collaborative Conference on Bioinformatics; 2009; Cleveland. p. 119-22

[103] Christiansen JK, Hughes JE, Welker DL, Rodriguez BT, Steele JL, Broadbent JR (2008). Phenotypic and genotypic analysis of amino acid auxotrophy in *Lactobacillus helveticus* CNRZ 32. Appl Environ Microb.

[104] Rahmen N, Fulton A, Ihling N, Magni M, Jaeger KE, Büchs J (2015). Exchange of single amino acids at different positions of a recombinant protein affects metabolic burden in *Escherichia coli*. Microb Cell Fact.

[105] Price MN, Zane GM, Kueh JV, Melnyk RA, Wall JD, Deutschbauer AM (2018). Filling gaps in bacterial amino acid biosynthesis pathways with high-throughput genetics. Plos Genet.

[106] Murcia R, Rodelas B, Salmeron V, Martinez-Toledo MV, Gonzalez-Lopez J (1997). Effect of the herbicide simazine on vitamin production by *Azotobacter chroococcum* and *Azotobacter vinelandii*. Appl Soil Ecol.

[107] Revillas JJ, Rodelas B, Pozo C, Martinez-Toledo MV, Lopez JG (2005). Production of amino acids by *Azotobacter vinelandii* and *Azotobacter chroococcum* with phenolic compounds as sole carbon source under diazotrophic and adiazotrophic conditions. Amino Acids.

[108] Ahumada-Manuel CL, Guzmán J, Peña C, Quiroz-Rocha E, Espín G, Núñez C (2017). The signaling protein MucG negatively affects the production and the molecular mass of alginate in *Azotobacter vinelandii*. Appl Microbiol Biot.

[109] Urtuvia V, Maturana N, Acevedo F, Peña C, Díaz-Barrera A (2017). Bacterial alginate production: an overview of its biosynthesis and potential industrial production. World J Microb Biot.

[110] Pacheco-Leyva I, Pezoa FG, Díaz-Barrera A (2016). Alginate biosynthesis in *Azotobacter vinelandii*: overview of molecular mechanisms in connection with the oxygen availability. Int J Polym Sci.

[111] Ertesvag H (2015). Alginate-modifying enzymes: biological roles and biotechnological uses. Front Microbiol.

[112] Ertesvåg H, Sletta H, Senneset M, Sun YQ, Klinkenberg G, Konradsen TA (2017). Identification of genes affecting alginate biosynthesis in *Pseudomonas fluorescens* by screening a transposon insertion library. BMC Genomics.

[113] Hay ID, Wang YJ, Moradali MF, Rehman ZU, Rehm BHA (2014). Genetics and regulation of bacterial alginate production. Environ Microbiol.

