# Supplementary material for: Yeast extracts from different manufacturers and supplementation of amino acids and micro elements reveal a remarkable impact on alginate production by A. vinelandii ATCC9046
Source: Microb Cell Fact. 2023 May 11;22:99. doi: 10.1186/s12934-023-02112-3 (PMC10176783; doi:10.1186/s12934-023-02112-3)
Supplement: Supplementary file 1 — Additional file 1: Table S1. Conditions to be selected to achieve desired final values for selected cultivation features. Figure S1. Oxygen transfer rates of microaerobic A. vinelandii ATCC9046 cultivations with two different yeast extracts. Figure S2. Effect of different yeast extracts on A. vinelandii ATCC9046 cultivations at sufficient oxygen supply. Figure S3. Acetylation degree and final broth viscosity of A. vinelandii ATCC9046 cultivations with different yeast extracts. Figure S4. Final broth viscosity of microaerobic A. vinelandii ATCC9046 cultivations with varying yeast extract concentrations. Figure S5. Further analytics of A. vinelandii ATCC9046 cultivations, supplemented with vitamins, micro elements and trace elements. Figure S6. Further analytics of A. vinelandii ATCC9046 cultivations with supplementation of copper, zinc and manganese sulfate. Figure S7. Further analytics of microaerobic A. vinelandii ATCC9046 cultivations with supplementation of different grouped amino acids. Figure S8. Further analytics of microaerobic A. vinelandii ATCC9046 cultivations with supplementation of single amino acids. Figure S9. Further analytics of A. vinelandii ATCC9046 cultivations with supplementation of micro elements and amino acids. [file 12934_2023_2112_MOESM1_ESM.docx]

**Additional File 1: Additional Tables & Figures**

**Manuscript:**

**Yeast extracts from different manufacturers and supplementation of amino acids and micro elements reveal a remarkable impact on alginate production by *A. vinelandii* ATCC9046**

**Sarah Sparviero^a^, Max Daniel Dicke^a^, Tobias M. Rosch^a^, Tania Castillo^b^, Holjes Salgado-Lugo^b,c^, Enrique Galindo^b^, Carlos Peña^b^ and Jochen Büchs^a,*^**

^a^ Aachener Verfahrenstechnik – Biochemical Engineering, RWTH Aachen University, Forckenbeckstr. 51, 52074 Aachen, Germany

^b^ Departamento de Ingeniería Celular y Biocatálisis, Instituto de Biotecnología, UNAM, Universidad Nacional Autónoma de México, Ave. Universidad 2001, Col.

Chamilpa, Cuernavaca, 62210, Morelos, México

^c^ Programa Investigadoras e Investigadores por México del CONACyT, Consejo Nacional de Ciencia y Tecnología, 03940, México City, México

^*^ Corresponding author:

Prof. Dr.-Ing. Jochen Büchs, RWTH Aachen University, Aachener Verfahrenstechnik – Chair of Biochemical Engineering, Bldg. NGP^2^, Forckenbeckstr. 51, 52074 Aachen, Germany; Phone: +49 (0) 241 – 80-24633; Fax: +49 (0) 241 80 22635; E-mail: jochen.buechs@avt.rwth-aachen.de, ORCID iD: 0000-0002-2012-3476

**Table S1: Conditions to be selected to achieve desired final values for selected cultivation features.** For the compilation, all conditions investigated in this work were compared and the respective values were chosen. Cultivations were conducted in modified Burk’s medium with 20 g/L sucrose and 68 mM MOPS-buffer. Standard deviations were calculated for n ≥ 3. Cultivation conditions: 250 mL shake flasks, initial pH = 7.2, filling volume V_L_= 50 mL, temperature T = 29 °C, shaking frequency n = 165 rpm, shaking diameter d_0_ = 50 mm.

| **Desired feature** | **Condition to choose** | **Final value** |
| --- | --- | --- |
| Highest cell dry weight | 6.00 g/L Merck yeast extract | 9.49 ± 1.04 |
| Highest alginate dry weight | 3.00 g/L Roth yeast extract + cysteine | 7.72 ± 3.35 |
| Highest viscosity | 4.50 g/L AppliChem yeast extract | 78.71 ± 5.89 |
| Highest mean molecular weight | 3.00 g/L Roth yeast extract + amino acid group 2 | 1652.33 ± 108.10 |
| Highest acetylation degree | 2.25 g/L Roth yeast extract | 4.60 ± 0.11 |





**Figure S1:** **Oxygen transfer rates of microaerobic *A. vinelandii* ATCC9046 cultivations with two different yeast extracts.** Cultivations were conducted in modified Burk’s medium with 3 g/L yeast extract ((A) from Roth Batch 1, (B) from AppliChem), 20 g/L sucrose and 68 mM MOPS-buffer. The oxygen transfer rate (OTR) was determined using a TOM device from Kuhner Shaker GmbH as well as a comparable in-house build RAMOS device. For clarity, only every fifth measuring point is shown. The various closed symbols each stand for separate experiments. For some experiments, duplicates are available, which are represented by corresponding open symbols. In total, 12 experiments with a total of 18 replicates with Roth Batch 1 yeast extract and 7 experiments with a total of 13 replicates with AppliChem yeast extract were conducted. These experiments were performed within a time span of 13 months. The mean values as well as the standard deviation can be seen in Figure 1 for cultivations with Roth Batch 1 and in Figure 3B for cultivations with AppliChem. The horizontal dashed line depicts the OTR value of 2 mmol/L/h for an easier comparison of the nominal cultivation time. Cultivation conditions: 250 mL shake flasks, initial pH = 7.2, filling volume V_L_= 50 mL, temperature T = 29 °C, shaking frequency n = 165 rpm, shaking diameter d_0_ = 50 mm.





**Figure S2: Effect of different yeast extracts on A. vinelandii ATCC9046 cultivations at sufficient oxygen supply.** Cultivations were conducted in modified Burk’s medium with 3 g/L yeast extract from different manufacturers, 20 g/L sucrose and 68 mM MOPS-buffer. (A), (B), (C): The oxygen transfer rate (OTR), the respiratory quotient (RQ), as well as the accumulated oxygen transfer (AOT) were determined using a TOM device from Kuhner Shaker GmbH. For clarity, only every fifth measuring point of the OTR and RQ curves is shown. The number of replicates is given with n; if n = 2, the mean value is shown and the shadow depicts the difference of the two replicates. In (B), the horizontal dashed line depicts an RQ of 1. (D): Final cell dry weight is given as mean value, calculated from six replicates. Final broth viscosity was measured in quadruplicates at a shear rate of 316 1/s. (E): The final broth viscosity as a function of shear rate was measured in given replicates. The shear rate of 316 1/s is illustrated in (E) by a vertical dashed line. Standard deviations were calculated for n ≥ 3 and are represented by shadows or error bars. For the cell dry weight and the viscosity in panel (D), no significant differences between the values could be detected. Cultivation conditions: 250 mL shake flasks, initial pH = 7.2, filling volume V_L_= 10 mL, temperature T = 29 °C, shaking frequency n = 350 rpm, shaking diameter d_0_ = 50 mm.





**Figure S3: Acetylation degree and final broth viscosity of *A. vinelandii* ATCC9046 cultivations with different yeast extracts.** Cultivations were conducted in modified Burk’s medium with 3 g/L yeast extract from different manufacturers, 20 g/L sucrose and 68 mM MOPS-buffer. Cultivations were conducted under microaerobic conditions. Standard deviations were calculated for n ≥ 3 and are represented by shadows or error bars. Standard deviations are in some cases so small, that they are not well recognizable. (A) The acetylation degree is given as mean values, calculated from at least five replicates. (B) The final broth viscosity as function of the shear rate (100 to 5000 1/s) was measured in given replicates. The shear rate of 316 1/s is illustrated by a vertical dashed line. These viscosities at a shear rate of 316 1/s are plotted as Fig. 4H. Cultivation conditions: 250 mL shake flasks, initial pH = 7.2, filling volume V_L_= 50 mL, temperature T = 29 °C, shaking frequency n = 165 rpm, shaking diameter d_0_ = 50 mm.





**Figure S4: Final broth viscosity of microaerobic *A. vinelandii* ATCC9046 cultivations with varying yeast extract concentrations.** Cultivations were conducted in modified Burk’s medium with yeast extract at different concentrations from three different manufacturers (A: Roth Batch 1 (R-1), B: AppliChem (AC), C: Merck (M)), 20 g/L sucrose and 68 mM MOPS-buffer. Cultivations were conducted under microaerobic conditions. The final broth viscosity as function of the shear rate (100 to 5000 1/s) was measured in given replicates. Standard deviations were calculated for n ≥ 3 and are represented by shadows. Standard deviations are in some cases so small, that they are not well recognizable. The shear rate of 316 1/s is illustrated by a vertical dashed line. These viscosities at a shear rate of 316 1/s are plotted as Fig. 4I. Cultivation conditions: 250 mL shake flasks, initial pH = 7.2, filling volume V_L_= 50 mL, temperature T = 29 °C, shaking frequency n = 165 rpm, shaking diameter d_0_ = 50 mm.





**Figure S5: Further analytics of *A. vinelandii* ATCC9046 cultivations, supplemented with vitamins, micro elements and trace elements.** Cultivations were conducted under microaerobic conditions in modified Burk’s medium with 3 g/L yeast extract Roth Batch 1, 20 g/L sucrose and 68 mM MOPS-buffer. Standard deviations were calculated for n ≥ 3 and are represented by shadows or error bars. Significant differences are marked with asterisks, corresponding to the significance level (** for 0.001 < p < 0.01). (A), (B) The final mean molecular weight and the acetylation degree of the produced alginate are given as mean values, each calculated from at least six replicates. (C) The final broth viscosity as function of the shear rate was measured in given replicates. The viscosities at a shear rate of 316 1/s are plotted in Fig. 5E. The shear rate of 316 1/s is illustrated by a vertical dashed line. Cultivation conditions: 250 mL shake flasks, initial pH = 7.2, filling volume V_L_= 50 mL, temperature T = 29 °C, shaking frequency n = 165 rpm, shaking diameter d_0_ = 50 mm.





**Figure S6: Further analytics of *A. vinelandii* ATCC9046 cultivations with supplementation of copper, zinc and manganese sulfate.** Cultivations were conducted under microaerobic conditions in modified Burk’s medium with 3 g/L yeast extract Roth Batch 1, 20 g/L sucrose and 68 mM MOPS-buffer. The number of replicates is given with n; if n = 2, the mean value is shown and the shadow depicts the difference of the two replicates; if n = 3, the mean value is shown and the shadow / error bar depicts the standard deviation. No significant differences between the acetylation degree values could be detected (panel B). (A) For clarity, only every fifth point is shown. The RQ of 1 is depicted as a horizontal black dashed line. (B) The final acetylation degree is given as mean values, calculated from at least six replicates. (C): The final broth viscosity was measured in given replicates. The shear rate of 316 1/s is illustrated by a vertical dashed line. These viscosities at a shear rate of 316 1/s are plotted as Fig. 6D. Cultivation conditions: 250 mL shake flasks, initial pH = 7.2, filling volume V_L_= 50 mL, temperature T = 29 °C, shaking frequency n = 165 rpm, shaking diameter d_0_ = 50 mm.





**Figure S7: Further analytics of microaerobic *A. vinelandii* ATCC9046 cultivations with supplementation of different grouped amino acids.** Cultivations were conducted in modified Burk’s medium with 3 g/L yeast extract Roth Batch 1, 20 g/L sucrose and 68 mM MOPS-buffer. The composition of each amino acid group is specified in Table 2. Standard deviations were calculated for n ≥ 3 and are represented by shadows. (A), (B) For clarity, only every fifth measuring point is shown. The number of replicates is given with n; if n = 2, the mean value is shown and the shadow depicts the difference of the replicates. The horizontal black dashed line in (A) illustrates the RQ of 1. (C) The final broth viscosity was measured in at least triplicates per cultivation. The shear rate of 316 1/s is illustrated in (C) by a vertical dashed line. These broth viscosities at a shear rate of 316 1/s are plotted as Fig. 7E. Cultivation conditions: 250 mL shake flasks, initial pH = 7.2, initial pH for AA group 1 = 6.78, initial pH for AA group 2 & 4 = 6.95, filling volume V_L_= 50 mL, temperature T = 29 °C, shaking frequency n = 165 rpm, shaking diameter d_0_ = 50 mm.



**Figure S8: Further analytics of microaerobic *A. vinelandii* ATCC9046 cultivations with supplementation of single amino acids.** Cultivations were conducted in modified Burk’s medium with 3 g/L yeast extract Roth Batch 1, 20 g/L sucrose and 68 mM MOPS-buffer. Amino acids from group 1, 2 and 3 were chosen for supplementation. The composition of each amino acid group is specified in Table 2. Standard deviations were calculated for n ≥ 3 and are represented by shadows or error bars. Significant differences are marked with asterisks, corresponding to the significance level (* for 0.01 < p < 0.05, ** for 0.001 < p < 0.01 and *** for p < 0.001). (A) For clarity, only every fifth measuring point is shown. The number of replicates is given with n; if n = 2, the mean value is shown and the shadow depicts the difference of the two replicates. The horizontal black dashed line in (A) illustrates an RQ of 1. (B) The final broth viscosity was measured in given replicates. The shear rate of 316 1/s is illustrated in (B) by a vertical dashed line. These viscosities at a shear rate of 316 1/s are plotted as Fig. 8E. (C) The final acetylation degree of the produced alginate is given as mean values, calculated from at least six replicates. Cultivation conditions: 250 mL shake flasks, initial pH = 7.2, initial pH for supplementation of aspartate = 6.95, filling volume V_L_= 50 mL, temperature T = 29 °C, shaking frequency n = 165 rpm, shaking diameter d_0_ = 50 mm.

**

**

**Figure S9: Further analytics of *A. vinelandii* ATCC9046 cultivations with supplementation of micro elements and amino acids.** Cultivations were conducted under microaerobic conditions in modified Burk’s medium with 3 g/L yeast extract Roth Batch 1, 20 g/L sucrose and 68 mM MOPS-buffer. Standard deviations were calculated for n ≥ 3 and are represented by shadows. Standard deviations are in some cases so small, that they are not well recognizable. (A) For clarity, only every fifth measuring point is shown. The number of replicates is given with n; if n = 2, the mean value is shown and the shadow depicts the difference of the two replicates. The horizontal black dashed line in (A) depicts the RQ of 1. (B) The final broth viscosity was measured in given replicates. The shear rate of 316 1/s is illustrated in (B) by a vertical dashed line. These broth viscosities at a shear rate of 316 1/s are plotted as Fig. 9D. (C), (D): The final mean molecular weight and the acetylation degree of the produced alginate are given as mean values, calculated from at least six replicates. No significant differences could be detected between those values. Cultivation conditions: 250 mL shake flasks, initial pH = 7.2, filling volume V_L_= 50 mL, temperature T = 29 °C, shaking frequency n = 165 rpm, shaking diameter d_0_ = 50 mm.
